# Structure–Activity Relationship Study of Antimicrobial Peptide with Cross-Kingdom Activity

**DOI:** 10.1021/acs.biochem.6c00195

**Published:** 2026-06-04

**Authors:** Aparna Palakkurussi Rathessan, Fereshteh Ghazisaeedi, Krithika Unmesh, Pascal-Kolja Bingül, Johannes Kupke, Suvrat Chowdhary, Dennis Hanke, Maria Andrea Mroginski, Marcus Fulde, Beate Koksch

**Affiliations:** † Institute of Chemistry and Biochemistry, 9166 Freie Universität Berlin, Berlin 14195, Germany; ‡ Institute of Microbiology and Epizootics, Center of Infection Medicine, 9166 Freie Universität Berlin, Berlin 14163, Germany; § Institute of Microbiology, University of Veterinary Medicine Hannover, Hannover 30173, Germany; ∥ Veterinary Center for Resistance Research (TZR), Freie Universität Berlin, Berlin 14163, Germany; ⊥ Department of Chemistry, 26524 Technische Universität Berlin, Berlin 10623, Germany; # School of Veterinary Medicine, Institute of Veterinary Anatomy, Freie Universität Berlin, Berlin 14195, Germany

## Abstract

The widespread emergence of antibiotic-resistant pathogens poses a significant global health challenge and underscores the need for novel approaches to accelerate antimicrobial discovery. Antimicrobial peptides (AMPs) have gained attention as promising candidates due to their broad-spectrum activity, including efficacy against multidrug-resistant bacterial strains. SAJO-2, an antimicrobial peptide developed by Sarojini and colleagues, features a tryptophan zipper-like motif incorporating a central d-Phe-2-Abz unit, where 2-Abz functions as a conformationally constrained β-turn-inducing peptidomimetic scaffold. Modification of SAJO-2 in prior joint work from our groups through differential fluorination enhanced its antimicrobial potency; however, it also increased susceptibility to enzymatic digestion by β-trypsin. To address this limitation, the current research focuses on improving the overall efficacy of SAJO-2 through the incorporation of D-amino acids, beta backbone modifications, and a bulky pentafluorinated amino acid residue. All modified peptides exhibit resistance to enzymatic degradation, while antimicrobial activity was retained to differing degrees across organisms.

## Graphical abstract

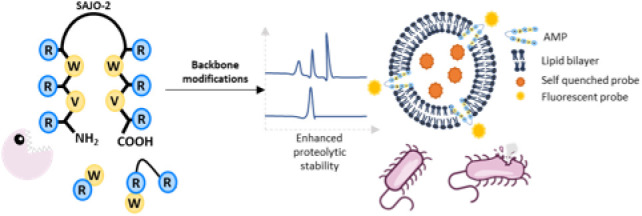

## Introduction

Antimicrobial resistance (AMR) is a critical global health concern that threatens the effective prevention and treatment of an ever-increasing range of infections caused by microbial pathogens.^1–4^ Ranked among the top 10 global health threats by the World Health Organization (WHO), AMR continues to challenge the effective treatment of common infections. A study assessing the worldwide burden of AMR from 1990 to 2021 reported 4.71 million deaths associated with bacterial AMR, of which 1.14 million deaths were directly attributable to bacterial AMR.^5^ Thus, there has long been a recognized need to develop novel antibiotics to maintain effective treatment options and ensure adequate access for the management of infectious diseases.

Antimicrobial peptides (AMPs), also known as host defense peptides (HDPs), are promising compounds that form an essential part of the innate immune system, providing broad-spectrum protection against invading microorganisms. They are found across all domains of life, from prokaryotes to eukaryotes.^6^ They have been increasingly recognized as potential lead structures for the design of next-generation antimicrobial agents aimed at overcoming antimicrobial resistance. Though diverse in length and structure, these peptides are generally amphipathic and positively charged, efficiently targeting Gram-negative and Gram-positive bacteria, viruses, fungi, and protozoa.^7–11^ They exert their activity through cationic residues, facilitating an electrostatic interaction with the negatively charged bacterial membrane, followed by the penetration of hydrophobic residues into the lipid tails, disrupting the bilayer structure and ultimately causing cell lysis.^12^ In 2023, Kakkerla et al. designed a library of peptides with varying properties and identified SK120 as a lead candidate, exhibiting potent *in vitro* antimicrobial activity and immunomodulatory effects with minimal hemolysis.^13^ Subsequent studies by Kavela et al. demonstrated the broad-spectrum efficacy of SK120 against multidrug-resistant bacterial strains, highlighting its potential as a scaffold for next-generation therapeutics.^14^ AMPs can adopt a multitude of biologically active conformations, with the most common being (i) α-helical, (ii) β-sheet, and (iii) extended/random coil structures.^15–17^ β-turns, which represent one of the most prevalent structural motifs in peptides and proteins, play a critical role in determining the specific β-hairpin conformation adopted,^18^ as these compact turns, typically consisting of 2–5 amino acid residues, facilitate the formation and stabilization of antiparallel β-strands commonly observed in bioactive peptides.

The β-turn motif d-Phe-2-Abz developed by Sarojini and coworkers as a synthetic mimic of the d-Phe-Pro turn in tyrocidine A, a membrane-lytic cyclic antimicrobial peptide,^19^ incorporates a sterically inflexible ortho-aminobenzoic acid (2Abz) moiety. Their amphipathic peptide SAJO-2 (named as peptide “2” in their work) represents a tryptophan zipper-like motif with an alternating arrangement of positively charged arginine residues and hydrophobic amino acids such as tryptophan and valine. Later in 2020, Varnava et al. reported that SAJO-2 having antibacterial and antifungal properties, exists as a mixture of conformers under physiological conditions, predominantly disordered with a minor β-hairpin population.^20,21^ Subsequent research by the Koksch group in 2023 investigated the potential of fluorinated amino acids to enhance efficacy; however, while all peptide analogues exhibit minimal hemolysis and low cytotoxicity toward HeLa and A549 cells, they are rapidly proteolytically degraded by β-trypsin.^22^

Our approach in this work was to employ a peptide backbone modification strategy to improve protease stability through the incorporation of noncanonical amino acids. Replacing L-amino acids with their d-enantiomers alters the peptide backbone’s stereochemistry and is a well-documented strategy in this respect.^23–26^ Moreover, β-amino acids, with an additional methylene group in the backbone, provide enhanced conformational flexibility and have been shown to protect peptides against enzymatic degradation.^27–29^ In addition, we incorporated a highly fluorinated residue, PfpGly, previously developed in our group^30^ to further increase hydrophobicity and enhance interaction with bacterial membranes. These modifications are recognized for influencing secondary structure formation, modulating membrane interactions, and extending biological half-life, making them promising strategies in the rational design and development of peptide-based therapeutics.

## Materials and Methods

### Chemicals and Reagents

All Fmoc-L-amino acids and D-amino acids for peptide synthesis were obtained from commercial suppliers (Carbolution, Sigma-Aldrich) and used without further purification, in accordance with the suppliers’ material safety data sheets. Beta-amino acids were purchased from abcr GmbH (Karlsruhe, Germany). Cl-MPA Protide resin and Fmoc-Arg (Pbf) Wang resin were supplied by CEM GmbH (Germany). The fluorinated amino acid PfpGly was synthesized according to a previously published protocol by our group.^30^ All solvents were obtained from Sigma-Aldrich and Fischer Scientific GmbH.

### Synthesis and Purification of Peptides

All peptides were synthesized with the microwave-equipped peptide synthesizer Liberty Blue (CEM, Matthews, NC, USA). All peptides were cleaved from the resin by treatment with a mixture of TFA/TIPS/H_2_O (90/5/5). A preloaded Fmoc-Arg (Pbf) Wang resin was used, and the synthesis was performed at a 0.1 mmol scale using Oxyma/DIC as activating reagents. Single coupling was performed for proteinogenic amino acids, while double coupling was used for the turn motif. Special coupling conditions were used for the incorporation of fluorinated amino acids.

Crude peptides were purified to >95% purity using reverse-phase high-performance liquid chromatography (RP-HPLC). Purification was performed using a Nexera Prep system from Shimadzu (Duisburg, Germany), which comprised a system controller (SCL-40), a UV–vis detector (SPD-40) with a 40UV flow cell of 10 mm path length, and a preparative liquid chromatograph (LC-20AP). A Kinetex C18 column (5 μM, 100 Å, 250 × 21.2 mm, Phenomenex, Torrance, CA, USA) was used. As eluents, water and ACN, both containing 0.1% (v/v) TFA, were applied. HPLC runs were performed with a flow rate of 15.0 mL/min, and UV detection occurred at 220 nm for the respective peptides. A linear gradient of 10–60% and 10–70% ACN + 0.1% TFA was utilized over 18 min. Data analysis was performed using EZChrom Elite software (Version 3.3.2 SP2, Agilent). Peptide purity was verified through analytical RP-HPLC employing a Chromaster 600 bar with a Kinetex C18 column. As eluents, ACN and H_2_O, both containing 0.1% (v/v) TFA was utilized. A flow rate of 1.0 mL/min was used, and the UV detection was monitored at 220 nm. The identity of the purified peptides was confirmed by an Agilent 6230 ESI-ToF MS instrument with a 1260 Infinity II LC system and a 1100 DAD detector (Figures S16–S29).

### Exchange of TFA Salts

Exchange of TFA salts was conducted according to a previously published protocol.^22^ Peptide samples were dissolved in a 5 mM HCl solution (5 mL) and stirred for 1 min at room temperature before lyophilization. This action was performed three times. To eliminate any remaining HCl, the peptide samples were then dissolved in Milli-Q water (10 mL) and stirred for 1 min before lyophilization. Analytical HPLC data and mass spectrometry data for all peptides can be found in the Supporting Information.

### Proteolytic Digestion Assay

The enzymatic digestion assay was performed based on a previously published protocol by our group.^22^ For real-time monitoring of enzymatic digestion for backbone-modified SAJO peptides, all peptide samples were dissolved in 10 mM phosphate buffer, pH 7.4 (170 μL, 100 μM peptide concentration), and gently mixed to obtain a homogeneous solution. Then, 35 μL of the enzymes (20 μM concentration in 10 mM phosphate buffer, pH 7.4) were gently mixed for 5 s. After that, the samples were incubated at 30 °C over a period of 3 h to 24 h. Aliquots of 25 μL were taken at fixed time points and quenched with 50 μL of a solution of 1% TFA in water containing 75 μM Ac-[2]­Abz-Gly-OH as a reference and 50 μL MeOH. Peptide degradation was monitored by HPLC analysis at regular time intervals. For determining the digestion profile of each AMP, all detected peptide fragments were isolated and identified through HRMS (Agilent 6230 System with 1260 Infinity II LC system and 1100 DAD detector) analysis. All experiments were performed in triplicate.

### Plasma Separation

Healthy porcine whole blood (2 mL) (provided by the Ruminant and Swine Clinic, Faculty of Veterinary Medicine, Freie Universität Berlin), anticoagulated with K2-EDTA, was centrifuged at 500*g* for 10 min at room temperature to isolate plasma without hemolysis. After centrifugation, the plasma was collected, and the cellular fraction was discarded.

### Plasma Stability Assay

The proteolytic stability of SAJO peptides against porcine blood plasma was analyzed based on a previously published protocol by our group.^31^ All peptide samples were dissolved in 10 mM phosphate buffer, pH 7.4 (170 μL, 100 μM peptide concentration) and incubated with 35 μL plasma solution at 37 °C over a period of 3 h. Aliquots of 25 μL were taken at regular time intervals, quenched with 100% ACN/0.1% TFA containing 75 μM Ac-[2]­Abz-Gly-OH as a reference peptide. The quenched samples were centrifuged at 6000 rpm for 2 min, and the supernatant solution was analyzed using analytical HPLC. Peptide fragments were isolated and detected through HRMS analysis. All experiments were performed in triplicate.

### Antimicrobial Susceptibility Testing

The minimum inhibitory concentration (MIC) of the peptides was determined by broth microdilution in cation-adjusted Mueller-Hinton Broth (MHII) using a flat-bottomed 96-well plates against the following microorganisms: *Escherichia coli* ATCC 25922 (standard strain in this study), Salmonella Typhimurium ATCC 14028, *Klebsiella pneumoniae* ATCC 700603, *Pseudomonas aeruginosa* ATCC 27853, *Staphylococcus aureus* ATCC 29213, *Enterococcus faecalis* ATCC 29212, and *Candida albicans* IMT 9655. MIC testing with *candida* strain were performed using the standard RPMI 1640 medium. Bacterial colonies were grown on blood agar or LB agar plates overnight at 37 °C. For antimicrobial testing, colonies were resuspended in MHII, and the optical density at 600 nm was measured. The bacterial suspension was diluted immediately before the experiment to achieve a concentration of 2 × 10^6^ CFU/mL. From a 2.5 mg/mL peptide stock solution in PBS, a starting concentration of 2048 μg/mL in MH II was prepared. Peptide concentrations ranging from 0.5 μg/mL to 1024 μg/mL were obtained by 2-fold serial dilution in the 96-well plate. To each well containing 50 μL of peptide solution in PBS/MHII, 50 μL of bacterial suspension was added. Wells containing medium only served as negative controls, while wells containing bacterial suspension without peptide served as positive controls, respectively. The OD at 460 (assay internal control) and 600 nm (bacterial growth measurement) was measured at time zero immediately after inoculation, and again after 24 h of incubation at 37 °C. The MIC value was detected at the lowest peptide concentration at which no bacterial growth could be observed after incubation. All measurements were performed in three technical replicates and repeated in three independent experiments to ensure reproducibility.

### Lipid-Binding Assay

Peptide solutions were prepared by 2-fold serial dilution. In a black 96-well plate, 50 μL of lipopolysaccharide from Salmonella Typhimurium in 50 mM Tris buffer, pH 7 and 50 μL of Bodipy-TR-cadaverine (in 50 mM Tris buffer, pH 7) were combined and stirred in the dark for 30 min. A positive control (F_max_) for maximum intensity was created using only Bodipy (excluding LPS and AMP), while a negative control (F0) consisted of Bodipy and LPS (excluding AMP). Subsequently, 100 μL of peptides at various concentrations were introduced to the LPS and Bodipy and stirred in the dark for 15 min. Following the incubation, fluorescence measurements were performed with an Infinite M Nano+ plate reader (Tecan Deutschland GmbH, Crailsheim, Germany) at 580 nm for excitation and 620 nm for emission. All measurements are conducted three times using three biological replicates.

The following formula was used to calculate BODIPY-TR-cadaverine displacement rates from LPS

$$\left(F_{\mathrm{compound}}-F_{0}\right)/\left(F_{\max}-F_{0}\right)\times 100$$

F_0_ is the fluorescence intensity of BODIPY-TR-cadaverine with LPS

F_max_ is the fluorescence intensity of BODIPY-TR-cadaverine without LPS

F_compound_ is the fluorescence intensity of the AMPs

Percentage displacement versus peptide concentration was plotted.

### 6-FAM Membrane Leakage Assay

Membrane permeabilization of SAJO peptides through the carboxyfluorescein leakage assay was conducted based on a previously published protocol by our group.^22^ Concentrated stocks (each 10 mg/mL) of 1-palmitoyl-2-oleoyl-*sn*-glycero-3-phosphoethanolamine (POPE) and 1-palmitoyl-2-oleoyl-*sn*-glycero-3-[phosphor-rac-(1-glycerol)] (POPG) were prepared by dissolving the compounds in CHCl_3_. Aliquots from both stocks were mixed, and CHCl_3_ was evaporated. The lipid film was dried in vacuo overnight and then dissolved in 50 mM 6-carboxyfluorescein (6-FAM) in 10 mM phosphate buffer, pH 7.4. The suspension was first treated with 10 freeze–thaw cycles, then ultrasonicated at rt for 30 min, and finally rested for 1 h at rt. Untrapped 6-FAM was removed by gel filtration on a PD-10 desalting column packed with Sephadex G-25. For each sample, 25 μL of peptide solution in serial dilutions was slowly added to 25 μL liposome solution. The final lipid concentrations were 5 mM POPE/POPG (1:1, each 2.5 mM). The mixed samples were incubated for 30 min at 30 °C before each measurement. 6-FAM leakage was detected by measuring the fluorescence intensity (λ_ex_ = 493 nm/ λ_em_ = 517 nm) with an Infinite M Nano+ plate reader (Tecan Deutschland GmbH, Crailsheim, Germany). 100% dye release was achieved through the addition of 5% (v/v) Triton X-100 in buffer. A negative control was constituted by measuring the fluorescence emission of the liposome solution containing only buffer.

### Cytotoxicity Assay

Cytotoxicity assays were performed using a CytoTox-ONE Homogeneous Membrane Integrity Assay (Promega). DMEM/F-12 supplemented with 10% v/v FBS was used for culturing the HeLa and A549 cells, and Iscove medium for THP-1 cells, which were seeded at 25% confluency in 96-well plates. These well plates were incubated at 37 °C and 5% CO_2_ until reaching approximately 85–90% confluency. The medium was exchanged for medium containing the AMPs in concentrations ranging from 32 μg/mL to 256 μg/mL and incubated for another 24 h. The well plates were equilibrated to RT, and 50 μL medium was removed from each well. One μL “Lysis solution” was added to the wells designated as positive controls to induce maximal cytotoxicity. Subsequently, 50 μL of CytoTox-ONE reagent was added to each well. The well plate was shaken for 30 s and left to incubate at RT for 10 min. After incubation, 25 μL of “Stop solution” was added. The well plate was shaken for an additional 10 s prior to the measurement of the fluorescence intensity (λ_ex_ = 560 nm/ λ_em_ = 590 nm). For all three cell lines, experiments were performed in three biological replicates, each containing three technical replicates per condition per plate.

### Hemolysis Assay

First, 8 mL of whole porcine blood containing heparin as an anticoagulant (provided by the Ruminant and Swine Clinic, Faculty of Veterinary Medicine, Freie Universität Berlin) was centrifuged at 1000*g* for 5 min at RT to separate the erythrocytes from the serum and buffy coat. The erythrocyte fraction was washed three times with 10 mL PBS. One mL of pure RBC suspension was diluted with PBS to obtain a final concentration of 2% (v/v) RBC. The lyophilized peptides were dissolved in PBS to a concentration of 512 μg/mL. In a 96-well plate (polystyrene, nontreated; Corning, New York, USA), peptide solutions were prepared by 2-fold serial dilution in PBS to a final volume of 100 μL per well, yielding concentrations ranging from 16 μg/mL to 256 μg/mL. Subsequently, 100 μL of the 2% (v/v) RBC suspension was added to each well containing peptide samples.

Wells containing only 1% (v/v) RBC in PBS served as negative controls. Positive controls were prepared by adding 0.1% and 1% Triton X-100 to 1% (v/v) RBC suspensions. The plate was incubated at 37 °C for 45 min without agitation. Upon incubation, the plate was centrifuged at 3500*g* for 10 min at RT, and 100 μL of the supernatant was transferred to a new 96-well plate. The absorbance was measured at 540 nm, and the hemolytic activity was calculated as follows:

$$\mathrm{\% Hemolysis}=\left(\frac{A_{exp}-A_{PBS}}{A_{100\%}-A_{PBS}}\right)\times 100$$

where A_exp_ denotes experimental absorbance, A_PBS_ denotes negative control, A_100%_ denotes positive control

All experiments were conducted in three technical replicates and repeated in three independent biological replicates.

### Transmission Electron Microscopy

*E. coli* (ATCC 25922) was cultivated in LB medium and diluted to reach an OD of 0.4 at 600 nm for every sample. The bacterial suspension was treated with SAJO-2D and SAJO-PfpGly-1D in PBS to obtain final concentrations corresponding to 2 × MIC and 4 × MIC. The samples were incubated at 37 °C for 1 and 24 h. Bacterial suspensions without peptide treatment were prepared in parallel and served as negative controls. After incubation, the samples were centrifuged at 4000*g* for 5 min. The supernatant was discarded, and the pellet was resuspended in PBS to remove residual medium. Following another centrifugation and removal of the PBS wash solution, the bacterial pellets were fixed in Karnovsky fixative (7.5% glutaraldehyde and 3% paraformaldehyde in PBS) for 24 h at 5 °C. Samples were washed with 0.1 M cacodylate buffer and then incubated in 1% osmium tetroxide for 2 h. Samples were dehydrated in graded ethanol concentrations and embedded in a mixture of epoxy resin, DDSA softener, MNA hardener, and a DMP-30 catalyst. The resin was sliced into semithin sections using an ultramicrotome and stained according to a modified Richardson protocol.^32^ Ultrathin slices (80 nm) were placed on nickel grids and examined with a transmission electron microscope (TEM, JEOL, JEMXXX).

### Adaptive Laboratory Evolution

Adaptive laboratory evolution (ALE) experiments were performed in a flat-bottom 96-well plate (polystyrene, nontreated; Corning, New York, USA). Initially, eight samples containing 100 μL *E. coli* (ATCC 25922, starting concentration 1 × 10^6^ CFU/mL) in MH II were treated with SAJO-peptides in PBS at sub-MIC and 1/4 × MIC concentrations. Plates were incubated at 37 °C for 24 h, and bacterial growth was monitored by measuring optical density at 600 nm using a plate reader (Synergy HTX Multimode Reader (BioTek)). Subsequently, 20 μL of the grown culture was transferred into a new well containing 80 μL of fresh MH II and treated again with a 2-fold increased peptide concentration. This serial passaging procedure was repeated until a peptide concentration of 512 × MIC was reached.

### Bacterial DNA Isolation; Whole Genome Sequencing; Genome Analysis

Genomic DNA was isolated from the ancestral frozen stock and from the first culture directly obtained from the experimental-evolution frozen stock of the evolved strain without any intermediate passaging using standard procedure, following the Qiagen “Isolation of Genomic DNA from Bacterial Suspension Cultures” protocol with the QIAmp DNA Mini Kit. DNA concentration and purity were assessed using a NanoDrop 1000 spectrophotometer (Thermo Fischer Scientific, Waltham, MA, USA) and quantified with a QubitTM 2.0 fluorometer (Thermo Fischer Scientific, Waltham, MA, USA). DNA integrity was additionally verified by agarose gel electrophoresis (1% TBE, 80 V, 90 min).

For short-read whole-genome sequencing, 1 ng of purified genomic DNA was used to generate sequencing libraries with the Nextera XT DNA Library Preparation Kit (Illumina, Inc., San Diego, USA), following the manufacturer’s instructions. Sequencing was performed on an Illumina MiSeq platform using the MiSeq Reagent Kit v3, generating 2 × 300-bp paired-end reads in a 4-sample multiplex format. Raw Illumina reads were trimmed with Trim Galore v0.6.6 (RRID:SCR 011847) and quality-assessed using FastQC. De novo genome assembly was conducted with Unicycler^33^ v0.4.9 and SPAdes^34^ v3.15.5. The final sequence analyses were conducted using Geneious software version 11.1.5.

### SCAN-LAG

ScanLag^35,36^ assays were performed on cultures of the ancestral strain and on the first culture directly obtained from the experimental-evolution frozen stock of the evolved strain without any intermediate passaging to quantify bacterial single-colony lag times and growth rates, following the approach of Levin-Reisman et al. Briefly, bacterial cultures were diluted to ∼10^3^ CFU/mL, and 100 μL of the bacterial suspension was spread onto LB agar plates. After absorption, plates were covered with a sterile black felt cloth and placed on a flatbed scanner. Scanning was conducted at 33 °C, corresponding to the maximum temperature tolerated by the scanners, using the UnixScanningManager software (https://ithub.com/nirda/UnixScanningManager, commit 5d94cd3) on a Linux operating system (Ubuntu 20.04 LTS AMD 64). Following an initial 240 min delay, plates were scanned every 15 min for 24 h.

Image processing was performed in MATLAB R2020a using a custom script incorporating the NQBMatlab analysis toolbox (https://github.com/oferfrid/NQBMatlab/tree/V16, commit 65306ce). Detection errors were curated manually, and final appearance and growth metrics were exported to Excel. Colony appearance time (lag time) was defined as the time required to reach a detectable area of 10 pixels, while growth time was defined as the time interval required for colony expansion from 15 to 45 pixels.

### Molecular Dynamics Simulations

The dynamics of SAJO-2, SAJO-2D, SAJO-PfpGly-1D, and SAJO-PfpGly were simulated both in aqueous solution and in the presence of a mixed POPG/POPE membrane using the molecular dynamics (MD) protocols described below.

Peptide models of SAJO-2, SAJO-2D, SAJO-PfpGly-1D, and SAJO-PfpGly were constructed in GaussView using a beta-turn motif from PDB: 6ANM and AlphaFold-3-predicted N-terminal and C-terminal fragments. Stereochemical modifications and fluorinations were performed manually. Structures were optimized using the extended tight-binding semiempirical quantum mechanical GFN2-xTB method implemented in xTB (v6.6.0).^37^ Force field parameters were generated using AmberTools (GAFF and ff99SB with AM1-BCC charges), and systems were solvated in the TIP3P water model. Energy minimization was performed in AMBER22 using 5000 steps (2500 steepest descent followed by 2500 conjugate gradient) with a 12 Å nonbonded cutoff and positional restraints on the peptides. Equilibration was carried out with *pmemd* in four stages. First, systems were heated from 0 to 150 K over 1 ns in the NVT ensemble using Langevin dynamics (γ = 1.0 ps^–1^, 1 fs time step). Second, the temperature was raised from 150 to 300 K over 1 ns in the NPT ensemble (Berendsen barostat, 1 bar). The third and fourth stages consisted of two 5 ns NPT simulations at 300 K and 1 bar using a Langevin thermostat and Berendsen barostat. SHAKE constraints were applied to bonds involving hydrogen, and a 12 Å cutoff was used for nonbonded interactions. Production MD was performed at 310 K and 1 bar using a Langevin thermostat (γ = 1.0 ps^–1^) and Monte Carlo barostat for 300 ns with a 2 fs time step. SHAKE was applied to all bonds involving hydrogen. Coordinates were saved every 50 ps and energies every 2 ps. Temperature, pressure, and volume remained stable and within physiologically relevant ranges throughout the simulations, confirming the thermodynamic stability of the systems.

For the membrane-peptide simulations, 1:1 POPG/POPE bilayers were generated using CHARMM-GUI, and peptides were positioned within the membrane environment using VMD. All peptide-membrane simulations were performed in GROMACS 2024.2. Peptide coordinates and parameters originally prepared in AMBER ff99SB format were converted to GROMACS-compatible files using *ParmEd*, ensuring seamless integration of force-field parameters. Lipids, water, and ions were parametrized using AMBER force fields-Lipid21 for lipids and TIP3P for water, thereby maintaining a fully AMBER-consistent description across all components. Energy minimization was performed with 5000 steps of steepest descent using a force convergence threshold of 100.0 kJ/mol/nm. Each system was equilibrated with positional restraints of 1000 kJ/mol/nm^2^ applied to the peptide. Temperature equilibration was carried out at 310 K using a V-rescale thermostat (coupling constant: 0.1 ps), followed by pressure equilibration at 1.0 bar with a Parrinello–Rahman barostat (time constant: 2.0 ps). Production simulations were run for 300 ns with a 2 fs time step using the leapfrog integrator and periodic boundary conditions. Long-range electrostatics were treated with the Particle Mesh Ewald method, and van der Waals interactions employed a 1.2 nm cutoff. This setup provided a stable and fully AMBER-consistent framework for analyzing peptide-membrane interactions.

Trajectory analysis was performed in VMD (v1.9.1) using the final 100 ns of the production runs. Custom Python scripts employing NumPy 1.20.3 and MDAnalysis 2.0.0 were used to compute RMSD, RMSF, and donor–acceptor distances.

## Results and Discussion

### Design and Synthesis of Amphipathic β-Hairpin Peptides

A series of amphipathic model peptides was designed and synthesized based on a d-Phe-2-Abz turn motif previously reported by Sarojini and coworkers (Table 1). The original peptide consists of a central d-Phe-2-Abz unit, wherein the β-turn is stabilized by three hydrogen bonds: (a) NH of residue I with CO of residue i+3 (2.90 Å); (b) NH of residue I with CO of residue i+2 (2.94 Å); and (c) intraresidue at i+2 (2.64 Å).^21^ This tryptophan zipper-like motif was constructed with an alternating sequence of positively charged arginine residues and hydrophobic amino acids, including tryptophan and valine. Subsequent research carried out on the Sarojini motif by our group involved substitution with amino acids fluorinated to varying degrees, and though these analogues demonstrated negligible hemolysis and reduced cytotoxicity against HeLa and A549 cells; unfortunately, they were shown to be rapidly degraded by β-trypsin.^22^

**Table 1:** Nomenclature of the SAJO Peptide Library, Where Amino Acids Are Represented by the Standard Three-Letter Code

Peptide	Sequence
SAJO-2	Arg-Val-Arg-Trp-Arg-[d-Phe-2-Abz]-d-Ala-Arg-Trp-Arg-Val-Arg
SAJO–D	d-Arg-Val-d-Arg-Trp-d-Arg-d-Leu-[d-Phe-2-Abz]-d-Ala-d-Arg-Trp-d-Arg-Val-d-Arg
SAJO-2LD	d-Arg-d-Val-d-Arg-d-Trp-d-Arg-d-Leu-[d-Phe-2-Abz]-d-Ala-d-Arg-d-Trp-d-Arg-d-Val-d-Arg
SAJO-1D	Arg-Val-d-Arg-Trp-Arg-d-Leu-[d-Phe-2-Abz]-d-Ala-Arg-Trp-d-Arg-Val-Arg
SAJO-2D	Arg-Val-d-Arg-Trp-d-Arg-d-Leu-[d-Phe-2-Abz]-d-Ala-d-Arg-Trp-d-Arg-Val-Arg
SAJO-PfpGly-1D	Arg-PfpGly-d-Arg-Trp-Arg-d-Leu-[d-Phe-2-Abz]-d-Ala-Arg-Trp-d-Arg-PfpGly-Arg
SAJO-1β	Arg-Val-βhomoarg-Trp-Arg-d-Leu-[d-Phe-2-Abz]-d-Ala-Arg-Trp-βhomoarg-Val-Arg
SAJO-2β	Arg-Val-βhomoarg-Trp-βhomoarg-d-Leu-[d-Phe-2-Abz]-d-Ala-βhomoarg-Trp-βhomoarg-Val-Arg

In the current work, we modified the backbone of the SAJO-2 peptide with D- and β-amino acids and introduced pentafluoropropylglycine (PfpGly) as a side chain. Table 1 presents the nomenclature and sequences for the designed peptide library.

### Proteolytic Digestion Assay

Initially, we investigated the stability of all peptides toward proteolytic degradation. The enzymes assayed, and their cleavage specificities, are as follows: β-trypsin cuts at Arg/Lys, α-chymotrypsin at the aromatics Phe/Trp/Tyr, Proteinase K digests a broad range of substrates, and carboxypeptidase B cleaves off C-terminal Arg/Lys residues. Results of digests of SAJO–D and SAJO-2D by trypsin and chymotrypsin are shown in Figure 1a–d, and all other digestion data are shown in Figures S1–S6. Satisfyingly, and in contrast to the SAJO-2 (Figure S7) parent peptide,^22^ all D-amino acid-modified SAJO analogues showed complete resistance toward degradation even after long incubation times, the single exception being the removal of a terminal arginine residue of SAJO-1D and SAJO-2D by Proteinase K, observed after 24 h (Figure S1). β-modified peptides were found to be relatively susceptible to protease cleavage (Figures S3–S6).

**Figure 1:**
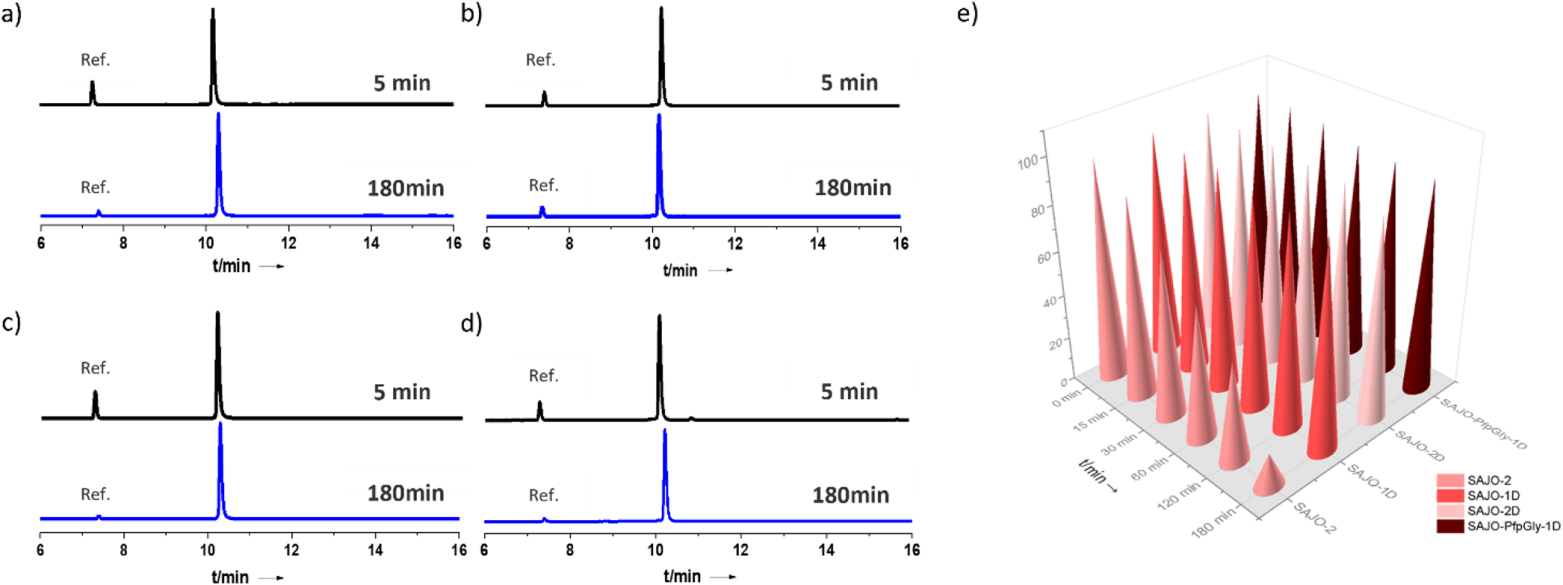
Real-time monitoring of enzymatic digestion of (a–b) β-trypsin and (c–d) α-chymotrypsin both at 20 μM final concentration, at time points 5 and 180 min (HPLC, DAD 280 nm) of SAJO–D and SAJO-2D, all at a 100 μM concentration during incubation at 30 °C. The dipeptide Ac-[2]­Abz-Gly-OH was used as a reference. (e) Real-time monitoring of the digestion of SAJO-2, SAJO-1D, SAJO-2D, and SAJO-PfpGly-1D (all at a 100 μM concentration) by porcine blood plasma at 0, 15, 30, 60, 120, and 180 min time points (HPLC, DAD 280 nm). All measurements were performed in triplicate.

Plasma stability assays (Figure 1e) also demonstrated that whereas the parent peptide SAJO-2 displayed rapid digestion, SAJO-1D, SAJO-2D, and SAJO-PfpGly-1D exhibited remarkable stability throughout even extended incubation periods. Thus, the proteolytic stability assays revealed that the incorporation of D-amino acids conferred substantial resistance to enzymatic degradation, whereas β-amino acid-modified SAJO analogues were susceptible to rapid degradation. Consequently, subsequent biological characterization was focused only on the D-modified SAJO peptides.

### Antimicrobial Susceptibility Testing

Minimum inhibitory concentration (MIC) determination is an essential method for assessing the susceptibility and resistance of pathogenic microbes to antimicrobial agents. Accordingly, the antimicrobial activity of the peptides was evaluated against strains of the following bacterial species (Table 2): *Escherichia coli* (Gram-negative), *Staphylococcus aureus* (Gram-positive), Salmonella Typhimurium (Gram-negative), *Pseudomonas aeruginosa* (Gram-negative), *Enterococcus faecalis* (Gram-positive), and *Klebsiella pneumoniae* (Gram-negative). In addition, the clinically relevant fungal pathogen *Candida albicans* was included. The MIC data demonstrate that D-amino acid substitution significantly influences the antimicrobial activity across the tested species. For *E. faecalis* and *P. aeruginosa*, most D-amino acid-modified peptides exhibited low potency (>1024 μg/mL), but SAJO-PfpGly-1D retained activity comparable to the parent SAJO-2 against *E. faecalis* (MIC 128 μg/mL). Regarding antifungal activity, the D-analogues maintained MIC values comparable to SAJO-2 against *C. albicans*. Compared with the control peptide, D-amino acid-modified analogues displayed comparable activity against *E. coli* and *S.* Typhimurium with MIC values in the range of 16–32 μg/mL. On the contrary, SAJO-2 and SAJO-PfpGly-1D also showed similar values (MIC 32 μg/mL) against *S. Typhimurium* and (MIC 64 μg/mL) against *K. pneumoniae* and *S. aureus*, while the remaining modified analogues demonstrated undesired 3-fold or 4-fold increase in MIC value.

**Table 2:** Antimicrobial Activities of Modified SAJO Peptides and Controls

	1*MIC [μg/mL]						
	Gram-negative bacteria				Gram-positive bacteria		Fungi
Peptide	*E. coli* (ATCC 25922)	*S. typhimurium* (ATCC 14028)	*K. pneumoniae* (ATCC 700603)	*P. aeruginosa* (ATCC 27853)	*S. aureus* (ATCC 29213)	*E. faecalis* (ATCC 29212)	*C. albicans* (IMT 9655)
SAJO-2	16	32	64	64	64	128	32
SAJO–D	16	32	64	512	256	1024	32
SAJO-2LD	256	-	-	-	256	-	-
SAJO-1D	32	32	64	1024	128	1024	32
SAJO-2D	32	16	128	1024	256	1024	32
SAJO-PfpGly-1D	32	32	64	512	64	128	32
SAJO-1β	64	-	-	-	128	-	-
SAJO-2β	128	-	-	-	256	-	-
Meropenem	<0.5	-	<0.5	<0.5	<0.5	1–2	-
Fluconazole	-	-	-	-	-	-	<0.5

### Evaluation of Membrane-Disrupting and Lipid-Binding Properties

The ability of antimicrobial peptides (AMPs) to bind to lipopolysaccharides (LPS), a key structural element of the outer membrane of Gram-negative bacteria, suggests their potential to impair bacterial membranes and counteract host immune evasion strategies. This interaction is essential for initiating membrane permeabilization, affecting bacterial viability, and possibly influencing host immune responses.

To assess AMP-LPS interactions, a BODIPY-cadaverine (BC) displacement assay was established. This fluorescence-based technique enables the quantitative evaluation of AMP binding to LPS and offers a comparative understanding of AMP specificity for Gram-negative compared to Gram-positive bacterial membranes. BODIPY-TR-cadaverine is a fluorescent indicator that noncovalently binds to LPS, thereby quenching its fluorescence. When AMPs interact with LPS, they displace BODIPY-TR-cadaverine dye, resulting in dequenching and a measurable rise in fluorescence intensity.

The degree of fluorescence intensity indicates the affinity of AMP for LPS and, as a result, its capacity to interact with and disrupt the outer membrane of Gram-negative bacteria. This assay thus provides a functional evaluation of both LPS-binding affinity and membrane-disruptive ability.

Figure 2 shows an increase in fluorescence with increasing concentrations of the antimicrobial peptides. The SAJO-2 parent peptide exhibits a comparatively higher binding affinity than the modified analogues. SAJO–D, SAJO-1D, SAJO-2D, and SAJO-PfpGly-1D behave similarly with lower fluorescence displacement. Their fluorescence intensities plateau much earlier, around 200–400 μg/mL, indicating weaker LPS binding compared to SAJO-2 (for binding affinities of β-peptides, see Figure S8).

**Figure 2:**
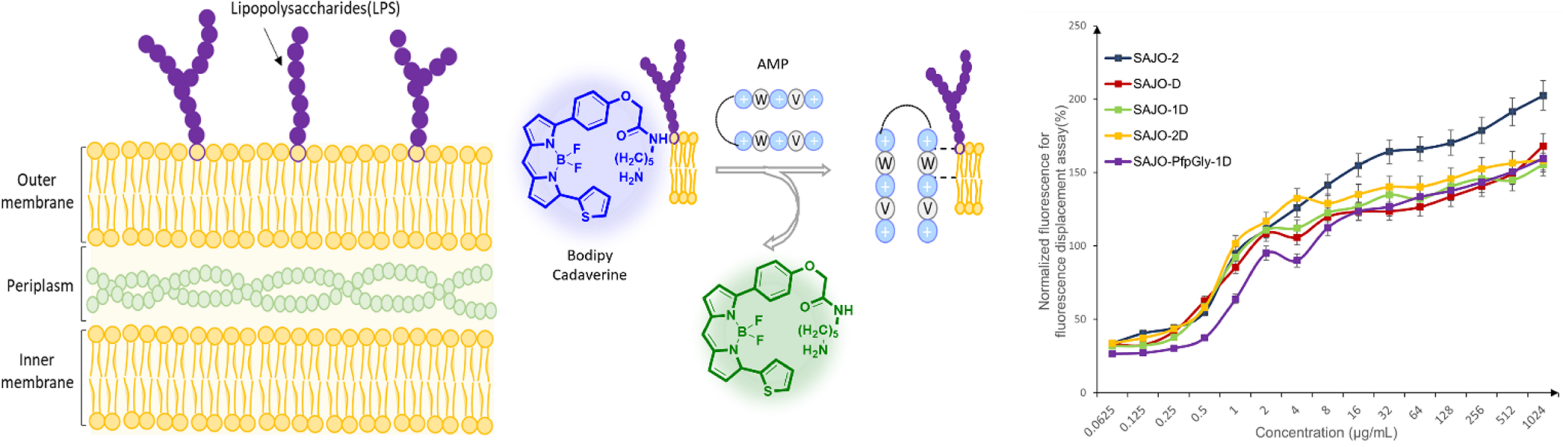
Lipid interaction of the peptides detected via the release of Bodipy TR cadaverine dye. Peptides (in 2-fold serial dilutions, 0.0625 μg/mL to 1024 μg/mL) were incubated with lipopolysaccharides from S. Typhimurium (7.5 μg/mL in Tris buffer) and Bodipy TR cadaverine (2.1 μM in Tris buffer). All experiments were performed with three technical replicates.

The activity of the peptides in permeabilizing membranes was further evaluated using a 6-carboxyfluorescein (6-FAM) leakage assay (Figure 3a). Large unilamellar vesicles (LUVs) made from equimolar proportions (1:1) of 1-palmitoyl-2-oleoyl-*sn*-glycero-3-phosphoethanolamine (POPE) and 1-palmitoyl-2-oleoyl-*sn*-glycero-3-phospho-(1’-rac-glycerol) (POPG) were infused with self-quenched 6-FAM. Peptide interaction disrupts the lipid bilayer, leading to dye release and a concentration-dependent increase in fluorescence. Fluorescence intensity was spectroscopically monitored and served as a direct measure of peptide-induced membrane lysis, thereby allowing the quantitative evaluation of lytic capability. Figure 3b shows dose–response curves that demonstrate similar membrane disruption for the parent peptide and all variants.

**Figure 3:**
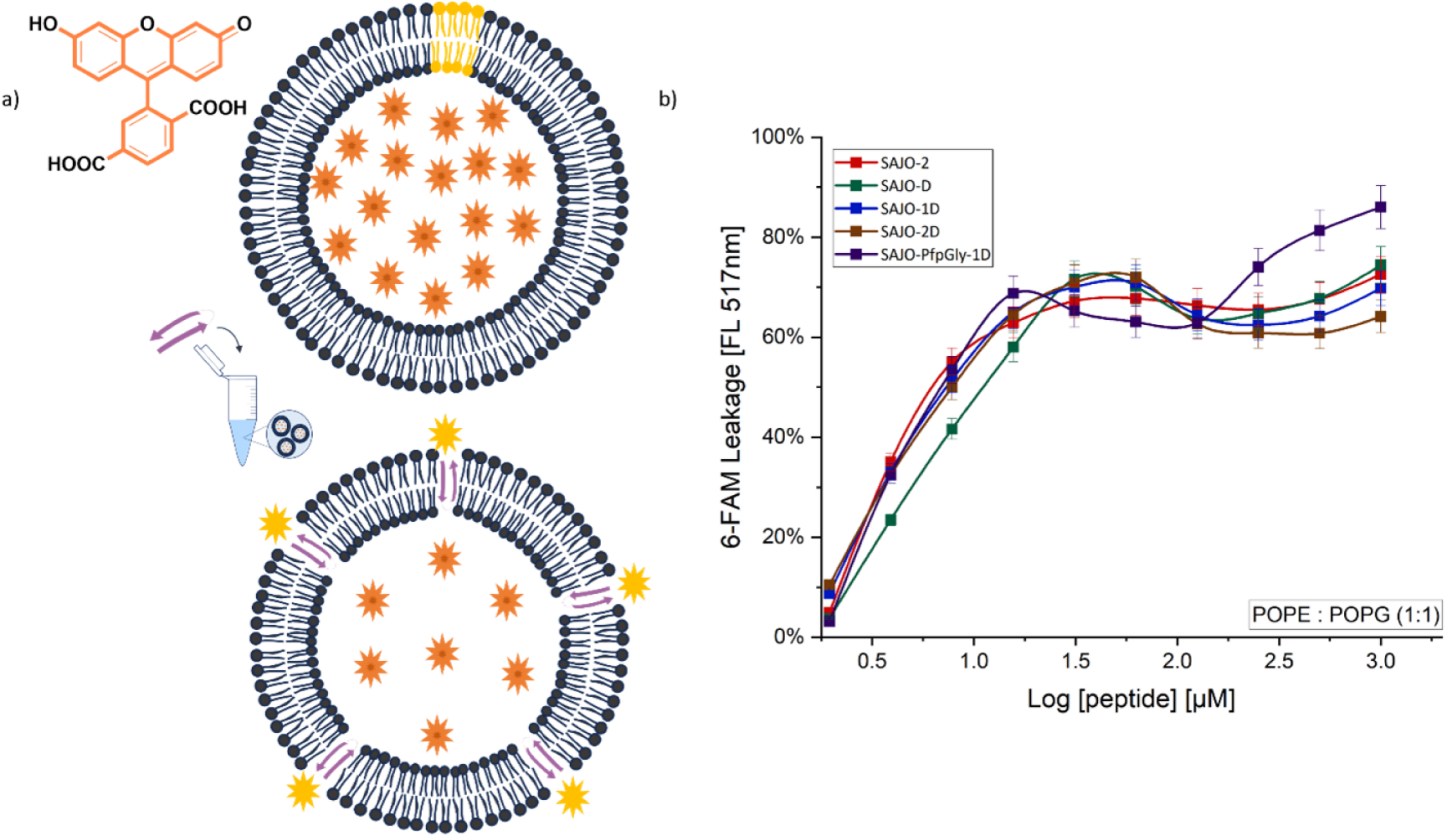
a) Graphical representation of membrane permeabilization assessed using a 6-FAM leakage assay. The dye was encapsulated within 5 mM liposomes composed of a 1:1 phospholipid mixture of POPE:POPG and its release was monitored by fluorescence spectroscopy (FL) (λex = 493 nm/λem = 517 nm) following incubation with peptides in serial dilutions at 30 °C for 30 min. FL values were normalized to 100% dye release obtained by treating liposomes with % (v/v) Triton X-100 as a positive control. b) Dose–response curves plotted from three separate measurements with the error bars showing standard deviation.

Furthermore, transmission electron microscopy (TEM) provided insights into the significant morphological alterations compared to the untreated control. While the negative control with the untreated *E. coli* demonstrated a uniform and dense cytoplasm with a defined cell wall (see Figure S9), the incubation of *E. coli* with the D-modified SAJO analogues showed significant membrane disruption, with uneven surfaces likely indicating membrane pore formation (see Figures S10–S11).

### Evaluation of Cytotoxic and Hemolytic Properties

The hemolytic activity of the peptides was evaluated by incubation with human red blood cells (Figure 4a). Across all peptides and concentrations tested, erythrocyte integrity remained above 95%, indicating negligible hemolysis. Notably, even at the highest tested peptide concentration (256 μg/mL), no significant hemolytic effects were observed. The absence of membrane-disruptive effects on RBCs indicates excellent hemocompatibility, a critical characteristic for systemically administered peptide therapeutics. These results suggest that the peptides exert their biological activity without disturbing the integrity of mammalian cell membranes.

**Figure 4:**
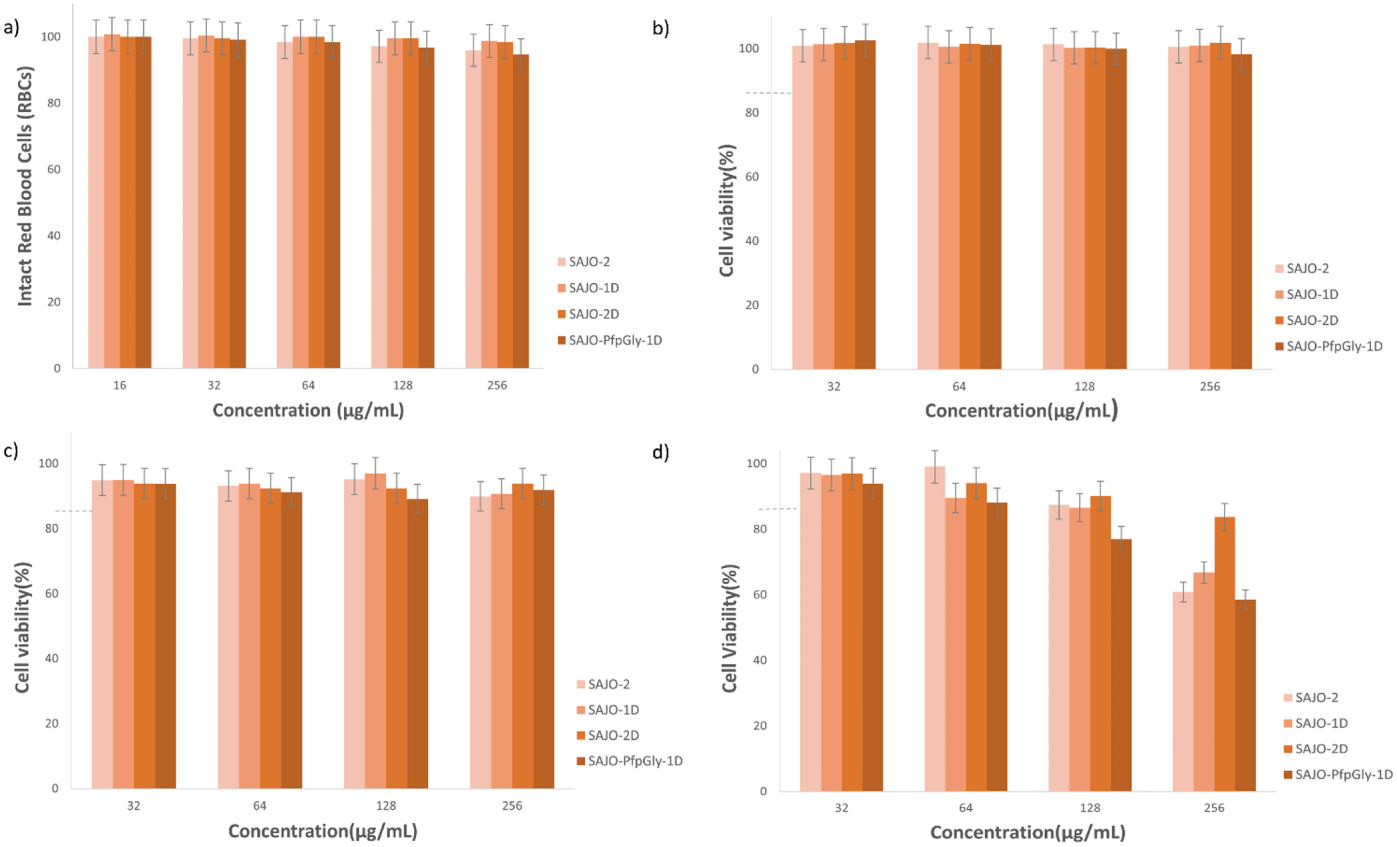
(a) Evaluation of peptide-induced hemolysis (incubation with 1% RBC solution) and (b-d) cytotoxicity with HeLa, A549, and THP-1 cell lines, respectively, for peptides SAJO-2, SAJO-1D, SAJO-2D, and SAJO-PfpGly-1D. Measurements were done in three technical replicates for each of three biological replicates.

Cytotoxicity was assessed using two epithelial cell lines (HeLa and A549) and THP-1 macrophages at peptide concentrations ranging from 32 to 256 μg/mL (Figure 4b–d). Consistent with the hemolysis data, incubation with SAJO-2, SAJO-1D, SAJO-2D, and SAJO-PfpGly-1D resulted in minimal cytotoxic effects in HeLa and A549 cells. Across all tested concentrations, cell viability remained between 90% and 100%, indicating good cellular tolerance and limited off-target toxicity, which is critical for therapeutic safety. Unlike polymyxins, one of the few remaining treatment options against multidrug-resistant Gram-negative infections, but whose clinical use is limited by nephrotoxicity and neurotoxicity,^38,39^ the SAJO peptides exhibit minimal hemolytic activity.

Conversely, a slight yet concentration-dependent cytotoxic effect was observed in the THP-1 cell line. At the highest concentration (256 μg/mL), SAJO-2 and SAJO-1D reduced cell viability to approximately 60–65%, while SAJO-PfpGly-1D exhibited a comparable decrease. Notably, SAJO-2D exhibited the lowest cytotoxicity at this concentration, with viability remaining above 85%. These results suggest differential cellular sensitivity among cell types and warrant further investigation into cell type-specific effects and the underlying mechanisms of action.

### Adaptive Laboratory Evolution Studies

The laboratory reference strain *Escherichia coli* ATCC 25922 was subjected to an experimental evolution regime involving stepwise increases in the concentrations of SAJO-2 and its two modified analogues, SAJO-2D and SAJO-PfpGly-1D, to assess and compare the adaptive evolutionary potential of bacterial populations exposed to these antimicrobial peptides (AMPs) (Figure 5). The evolution assay was initiated with a half-MIC concentration of SAJO-2 and a fourth MIC concentration of SAJO-2D and SAJO-PfpGly-1D. Over the 12-day selection period, populations repeatedly adapted to each peptide variant and successfully progressed to higher concentration steps. These observations highlight the remarkable evolutionary plasticity of bacteria.

**Figure 5:**
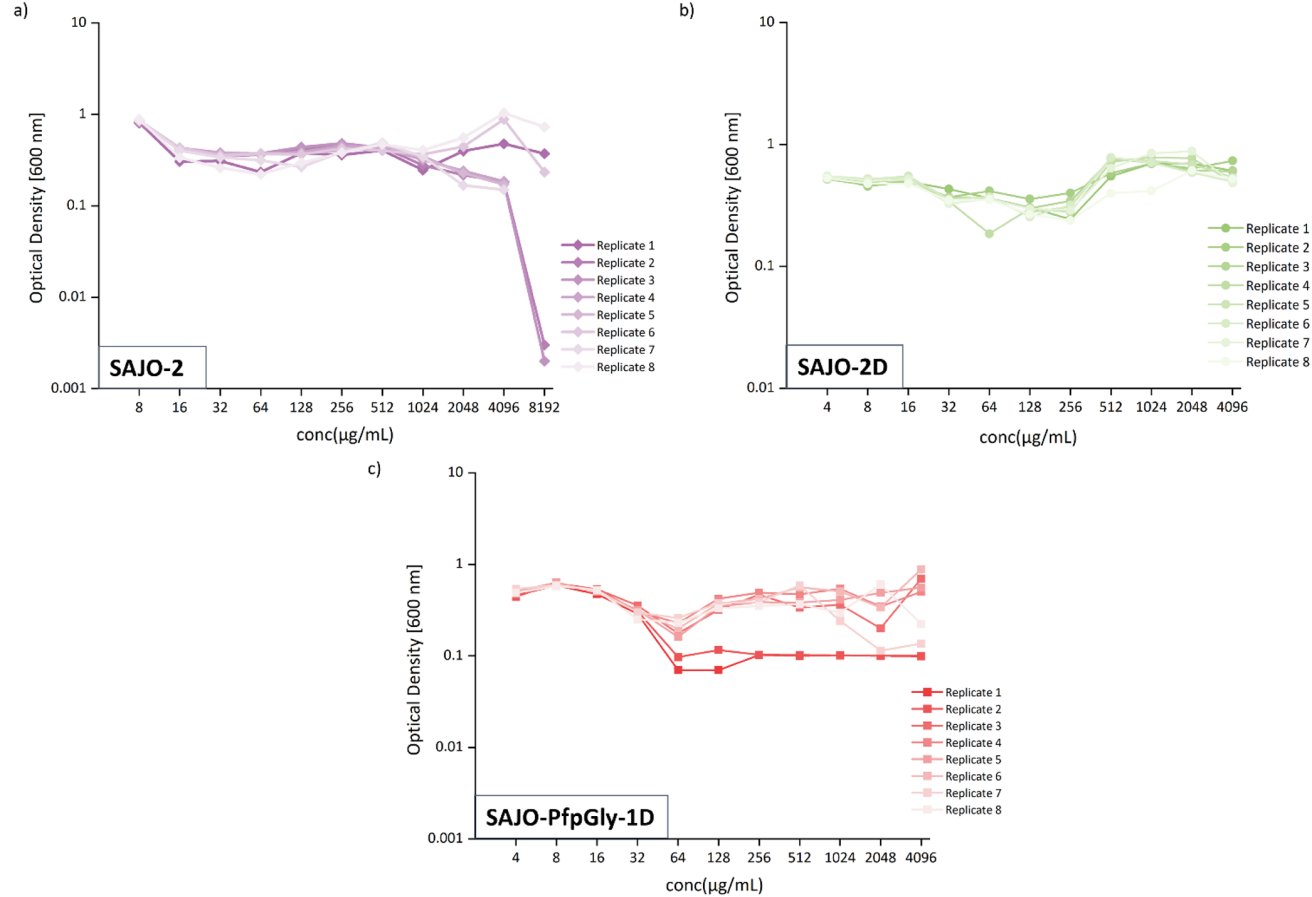
Adaptive evolution studies of a) SAJO-2, b) SAJO-2D, and c) SAJO-PfpGly-1D were conducted against *E. coli* (ATCC 25922) for 11 days with a daily 2-fold increase in peptide concentration. Experiments were performed using eight parallel replicates.

During evolution in the presence of SAJO-2D, the bacterial population not only exhibited elevated resistance (Table 3) but also developed pronounced colony-morphology heterogeneity, producing both small and large colonies (see Figure S12). Such phenotypic diversification—common in natural bacterial populations exposed to unfavorable conditions^40,41^ —reflects the coexistence of multiple survival strategies that increase the likelihood to persist under stressful conditions.

**Table 3:** Antimicrobial Activities of SAJO-2, SAJO-2D, and SAJO-PfpGly-1D Against Evolved Strains of *E. coli*

1* MIC [μg/mL]			
Bacterial strains	SAJO-2	SAJO-2D	SAJO-PfpGly-1D
*E. coli* (WT)	16	16	32
SAJO-2_Day11_evolved strain A	256	-	-
SAJO-2_Day11_evolved strain H	256	-	-
SAJO-2D_Day11_evolved strain A	-	1024	-
SAJO-2D_Day11_evolved strain B (Small colonies)	-	1024	-
SAJO-2D_Day11_evolved strain B (Big colonies)	-	1024	-
SAJO-PfpGly-1D_Day11_evolved strain F	-	-	128
SAJO-PfpGly-1D_Day11_evolved strain C	-	-	512

This phenotypic heterogeneity can arise from a variety of genetic and nongenetic mechanisms. In addition to stable genetic mutations or acquisition of resistance genes via horizontal gene transfer, bacteria can circumvent antibiotic pressure by nongenetic strategies, such as extended lag times and reduced metabolic activity, observed in antibiotic persistence and tolerance.^42^ Another mechanism employed by bacteria to combat antibiotics can be so-called adaptive resistance that underlies no inherited genetic alterations but inducible resistance via external stress signals.^43^ Epigenetic regulation has also been shown to drive the emergence of hypervirulent colony variants in *E. coli*.

Whole-genome sequencing (WGS) of the ancestral strain and evolved isolates representing the small- and large-colony subpopulations revealed no detectable stable genomic alterations such as SNPs (single nucleotide polymorphisms), as well as no transient repeated regions such as duplication-amplification events (GDA). The latter is the most common resistance mechanism in Gram-negative bacteria, causing heteroresistance, with a resistance factor only transiently present in subpopulations.^44,45^

The reversibility of the resistance phenotype in our study (Table S2) and the absence of SNPs or GDAs imply that the adaptive response is driven primarily by regulatory changes in gene expression. One possible explanation could be stressor-inducible two-component systems, which ultimately lead to changes in lipid A and resistance to antimicrobial peptides.^46^

In summary, these findings implicate that, upon exposure to SAJO-2D, bacteria initially adopt transient, mixed metabolic and/or growth strategies, similar to those described for noninherited antibiotic resistance. Under prolonged selective pressure, this mixed bacterial population may eventually accumulate stable genomic alterations, such as low-cost point mutations, resulting in permanent resistance to these AMPs. Indeed, evolution from unstable resistance phenomena to stable, genetically determined resistance has been observed for antibiotic tolerance as well as in heteroresistance.^47,48^

Additionally, minimum inhibitory concentrations (MICs) of the ancestral strain and representative evolved isolates at the experimental end point are presented in Table 3, demonstrating that *E. coli* rapidly adapts to the AMP-mediated selective pressure. Notably, as mentioned above, this resistance phenotype was reversible: following more than six passages in peptide-free medium, MIC values progressively declined toward the ancestral baseline (see Table S2).

Scan-Lag assays performed across multiple replicate plates with the ancestral strain and the two-colony morphology subpopulations of the evolved strain exposed to SAJO-2D confirmed that differences in colony-appearance times between the small- and large- colony variants represent genuine phenotypic distinctions and stochastic artifacts (see Figure S13), when considering the evolved population in contrast to the ancestral population. The evolved phenotypes exhibited a shorter lag time and an overall longer growth time.

### Molecular Dynamics Simulations

Molecular dynamics simulations of SAJO-2, SAJO-2D, and SAJO-PfpGly-1D in aqueous solution reveal distinct equilibrium structures and dynamic behaviors for the four peptides. Analysis of the root-mean-square deviation (rmsd) of the backbone carbon atoms of the ten lowest energy peptide structures relative to the minimum energy conformation (Figure 6) identified SAJO-2 with RMSD (2.82 ± 1.02 Å) as the least flexible peptide with a well-defined and stable zipper-like conformation in solution. In contrast, the introduction of D-amino acids at positions 3, 5, 9, and 11 in SAJO-2D disrupts this architecture, leading to a more open structure and a markedly broader conformational ensemble (RMSD of 5.10 ± 1.09 Å). This increased structural heterogeneity correlates with the peptide’s enhanced proteolytic resistance, which primarily arises from stereochemical mismatch: most proteases have chiral sites optimized to recognize and cleave L-configured substrates.^49^ Incorporation of fluorinated amino acid along with the addition of d-Arg decreases RMSD compared to SAJO-2D, as reflected by RMSD of 4.38 ± 0.7 Å, but does not restore its conformational stability as in SAJO-PfpGly-1D. For comparison, additional MD simulations of fluorinated SAJO but within all-L peptide (SAJO-PfpGly) were performed. Accordingly, SAJO-PfpGly does not bring about extensive conformational disorder as observed for D substitution, suggesting fluorination mainly fine-tunes the accessible conformations rather than increasing backbone stability.

**Figure 6:**
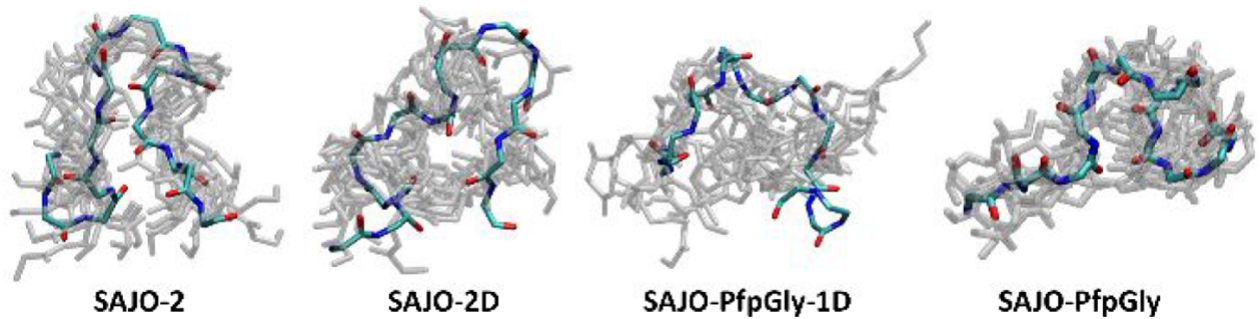
Conformational space of SAJO-2, SAJO-2D, SAJO-PfpGly-1D, and SAJO-PfpGly in aqueous solution as predicted by MD simulations. For clarity, only the peptide backbones of the ten lowest-energy conformers are shown in transparent gray licorice. The minimum-energy conformer is highlighted using standard atom coloring (C = cyan, O = red, *N* = blue).

Having characterized the conformational behavior of the peptides in aqueous solution, we next examined how their structures and dynamics are altered upon interaction with a 1:1 POPG/POPE lipid bilayer. For each of the three peptides (SAJO-2, SAJO-2D, and SAJO-PfpGly-1D), three independent peptide-membrane MD simulation replicates were generated. All three peptides exhibit substantial interactions with the membrane surface, as revealed by the contact frequency analysis shown in Figure S14. These plots quantify residue-lipid contacts using a 3 Å cutoff, reporting for each matrix element the percentage of simulation frames in which a given residue lies within this distance of a specific lipid headgroup. For SAJO-2, most residues show markedly stronger contacts with POPG (40–80%) than with POPE (<30%). The highest POPG interactions occur at the N-terminus—Arg3n (80%), Trp1n (77%), Arg2n (73%), and Arg1n (63%)—while Val1n displays a lower value (44%). Contact frequency gradually decreases toward the C-terminus, through Arg5c (68%) and Arg4c (63%) still interact prominently. The β-turn residues (Leu1, Phe1, 2-Abz, Ala1) moderate POPG contacts (34–60%), with Leu1 (60%) and Phe1 (53%) exhibiting the highest.

Similar to SAJO-2, SAJO-2D interacts predominantly with POPG. POPE contacts are largely restricted to terminal residues, while the β-turn exhibits little interaction. In contrast, POPG contacts are substantially higher, particularly at the C-terminus (Arg6c 94%, Arg4c 78%, Arg5c 75%), with Trp2c also contributing (62.4%). Overall, SJAO-2D shows stronger and more extensive membrane contacts than SAJO-2, especially through its C-terminal residues, and displays enhanced POPG selectivity. Among the three peptides, SAJO-PfpGly-1D displays the strongest minimal POPE interaction profile, followed by tryptophan and the PfpGly unit.

Beyond quantifying residue-lipid contacts, we next examined the binding geometries adopted during membrane association. For each frame of all simulations, the total peptide-membrane headgroup interaction energy (Coulombic + van der Waals) was computed and ranked, allowing us to identify the lowest-energy binding conformations for every replicate. Five lowest-energy structures from each trajectory were extracted (15 per peptide). Visual inspection revealed that the same set of residues consistently mediates binding, indicating robust and reproducible interaction motifs. For the discussion below, we therefore selected the single lowest-energy structure for each peptide. In SAJO-2 (Figure 7), binding is dominated by arginine side chains, which interact strongly with POPG via electrostatic attraction to the negatively charged phosphate groups, complemented by hydrogen bonding from the guanidinium moiety. Tryptophan also contributes substantially: its indole ring preferentially localizes at the lipid–water interface, and the N–H group forms hydrogen bonds with phosphate oxygens of POPG, consistent with its established anchoring role in membrane proteins. This interaction pattern explains the stronger displacement of BODIPY-cadaverine from LPS and the higher membrane permeabilization observed in the 6-FAM leakage assay for SAJO-2.

**Figure 7:**
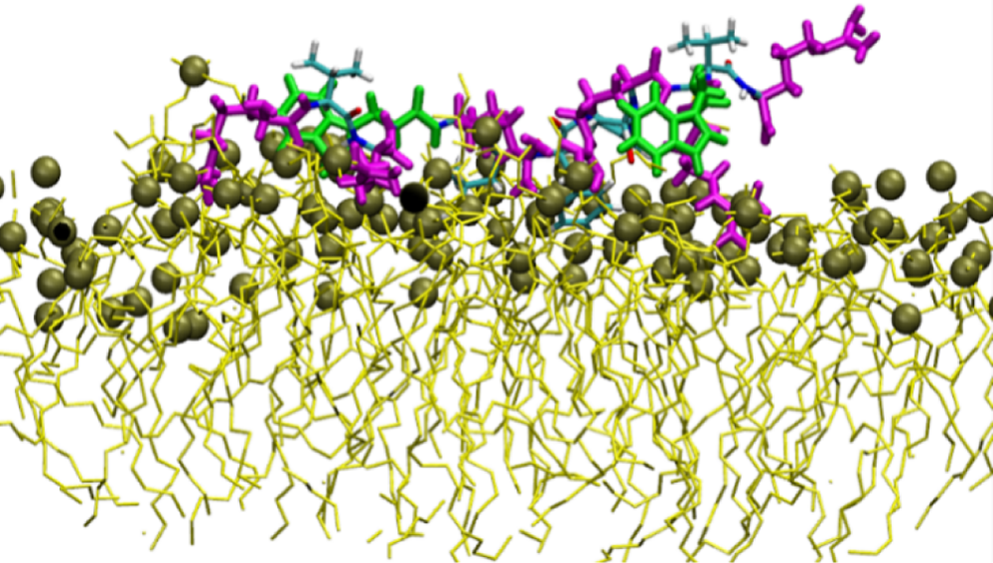
Representative binding poses of the SAJO-2 peptide at the POPG:POPE membrane. Arginine and tryptophan side chains are shown as magenta and green licorice, respectively, and lipid phosphate groups are displayed as gold spheres.

To further rationalize these binding differences, electrostatic surface potential maps of representative membrane-bound structures were analyzed (Figure 8). The spatial distribution of positive (blue) and neutral (white) regions closely mirrors the contact patterns. In SAJO-2, the main positively charged patch formed by arginine’s lies directly at the membrane interface, consistent with strong POPG engagement. In SAJO-2D and SAJO-PfpGly-1D, however, this positive patch progressively shifts outward, while a more neutral surface becomes exposed toward the membrane. As a consequence, the effective cationic region available for direct headgroup contacts is highest in SAJO-2 and increasingly electrostatically neutralized in SAJO-2D and SAJO-PfpGly-1D. These changes arise from different molecular origins. In SAJO-2D, inversion of stereochemistry alters the 3D arrangement of side chains, modifying the electrostatic surface and accounting for its enhanced POPG binding relative to SAJO-2. In SAJO-PfpGly-1D, fluorination locally changes polarity and polarizability, introducing regions of higher electron density and further shifting the electrostatic potential landscape—explaining the strongest membrane binding observed among the three peptides. Consistent with this picture, peptide insertion depths (Figure S15) increase from SAJO-2 to SAJO-2D to SAJO-PfpGly-1D, supporting the trend of progressively stronger electrostatic neutralization and membrane stabilization inferred from the potential maps.

**Figure 8:**
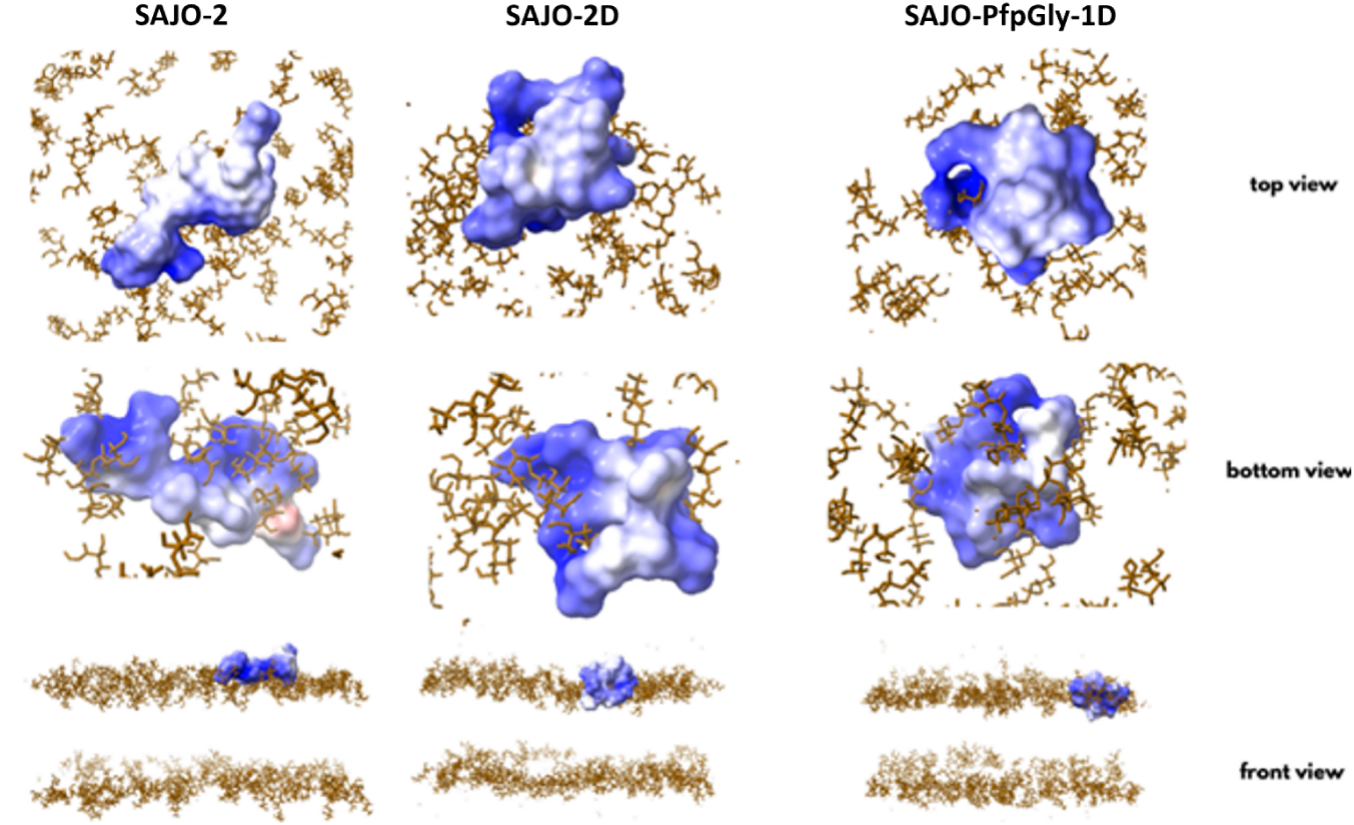
Top, bottom, and front views of representative SAJO-2, SAJO-2D, and SAJO-PfpGly-1D peptides bound to a 1:1 POPG:POPE membrane. Peptide surfaces are colored according to electrostatic potential (blue = positive, red = negative).

## Conclusion

In this work, we systematically substituted the L-amino acids in the SAJO peptide scaffold with their d-enantiomeric counterparts and other noncanonical amino acids, such as β-homoarginine, which incorporates an additional methylene group into the side chain, and a pentafluorinated amino acid, intending to increase the proteolytic stability without affecting the biological activity. All D-amino acid-incorporated SAJO analogues proved to be stable toward enzymatic degradation by the tested endo- and exoproteases, as well as porcine-derived blood plasma. MIC screenings revealed that the analogues largely retained antimicrobial activity against Gram-negative bacterial strains and the tested fungal *C. albicans* strain. In addition, all D-amino acid-modified peptides evaluated in this study were capable of binding to the lipid A in Gram-negative bacterial membranes and exhibited negligible hemolytic activity and low cytotoxicity. Furthermore, in an evolutionary experiment using an *E. coli* ATCC strain as a model organism, we observed rapid bacterial adaptation to AMPs, characterized by a transient resistance phenotype, suggesting a nonheritable resistance mechanism that appears to be conserved across multiple peptide analogues despite molecular modifications. Hence described, these findings underscore the immense potential of conformational tuning and the impact of fluorination in peptide architecture. Utilizing these analogues allowed us in this work to retain good cross-kingdom antibiotic activity and high proteolytic resistance, leading to promising therapeutic candidates, especially considering potential future combination treatments.

## Supplementary Material



## Notes

All experimental details for peptide synthesis, purification, and characterization are given, as well as all additional experimental procedures and figures for each approach and the techniques used (PDF)
