# Supplementary material for: Structure–Activity Relationship Study of Antimicrobial Peptide with Cross-Kingdom Activity
Source: Biochemistry. 2026 Jun 4;65(12):1944–56. doi: 10.1021/acs.biochem.6c00195 (PMC13276844; doi:10.1021/acs.biochem.6c00195)
Supplement: Supplementary file 1 [file bi6c00195_si_001.pdf]

SUPPLEMENTARY INFORMATION

Structure-activity relationship study of antimicrobial peptide with cross-kingdom activity

Aparna Palakkurussi Rathessan [1], Fereshteh Ghazisaeedi [2,3], Krithika Unmesh [5], Pascal-Kolja Bingül [6], Johannes Kupke [2], Suvrat Chowdhary [1], Dennis Hanke[2,3], Maria Andrea Mroginski [5], Marcus Fulde [2,3,4]*, Beate Koksch [1]*

A. P. Rathessan, Prof. Dr. B. Koksch
Institute of Chemistry and Biochemistry
Freie Universität Berlin
Arnimallee 20, 14195 Berlin, Germany

F. Ghazisaeedi, J. Kupke, D. Hanke, Prof. Dr. M. Fulde
Institute of Microbiology and Epizootics, Center of Infection Medicine
Freie Universität Berlin
Robert-von-Ostertag-Str. 7-13, 14163 Berlin, Germany
F. Ghazisaeedi, Prof. Dr. M. Fulde
Institute of Microbiology
University of Veterinary Medicine Hannover
Bischofsholer Damm 15, 30173 Hannover, Germany
Prof. Dr. M. Fulde
Veterinary Center for Resistance Research (TZR)
Freie Universität Berlin
Robert-von-Ostertag-Str. 7, 14163 Berlin, Germany

K. Unmesh, Prof. Dr. M. A. Mroginski
Department of Chemistry
Technische Universität Berlin
Straße des 17. Juni 115, 10623 Berlin, Germany

P. K. Bingül
School of Veterinary Medicine
Institute of Veterinary Anatomy
Freie Universität Berlin
Koserstrasse 20, 14195 Berlin, Germany

*Corresponding author:
Prof. Dr. Beate Koksch
beate.koksch@fu-berlin.de, Phone: +49 308 385 5344

Prof. Dr. Marcus Fulde
marcus.fulde@tiho-hannover.de, Phone: +49 511 856 8344

Table of Contents

1. Proteolytic digestion further data 3
2. Lipid binding assay further data 7
3. Transmission electron microscopy images 8
4. Adaptive laboratory evolution further data 10
5. Molecular dynamic simulations further data 11
6. Synthesis and purification of peptides 14
   6.1 SAJO-2 15
   6.2 SAJO-D 16
   6.3 SAJO-1D 17
   6.4 SAJO-2D 18
   6.5 SAJO-PfpGly-1D 19
   6.6 SAJO-β 20
   6.7 SAJO-2β 21
7. References 22

1. Proteolytic digestion further data

General conditions for proteolytic digestion have been provided in Table S1.

In Figures S3-S6, the digestion profiles of SAJO-β and SAJO-2β have been provided with corresponding HPLC chromatograms.

Table S1: HPLC gradient for digestion assays

Time [min]	Solvent A [%]	Solvent B [%]	Flow rate [mL/min]
0.0	90.0	10.0	1.00
18.0	30.0	70.0	1.00
19.0	0.0	100.0	1.00
21.0	0.0	100.0	1.00
22.0	90.0	10.0	1.00
25.0	90.0	10.0	1.00

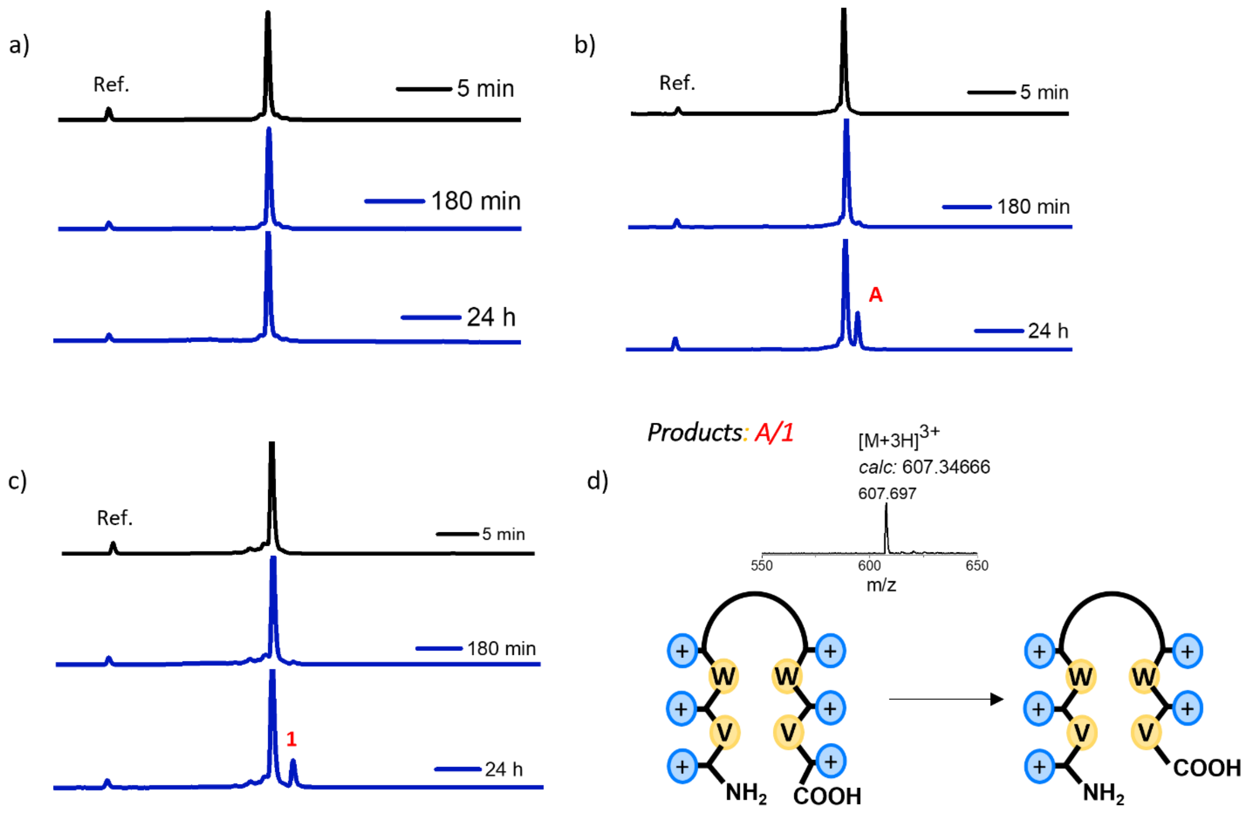

Figure S1: (a-c) HPLC chromatograms of **SAJO-D, SAJO-1D, SAJO-2D** (100 µM peptide with 20 µM **Proteinase K** in 10mM phosphate buffer, pH 7.4 at 30 °C). The dipeptide Ac-[2]Abz-Gly-OH was used as a reference signal. **(d)** MS-based detection and determination of peptide fragments of SAJO-1D and SAJO-2D. Measurements were performed in triplicate.

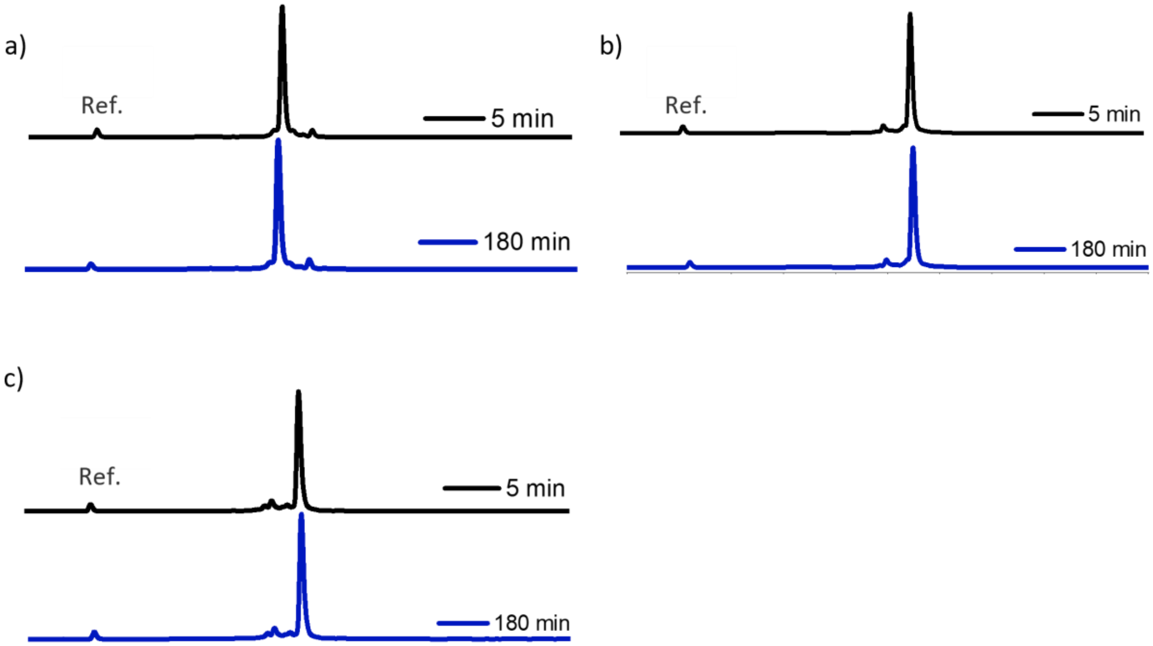

Figure S2: (a-c) HPLC chromatograms of **SAJO-D, SAJO-1D, SAJO-2D** (100 µM peptide with 20 µM **Carboxypeptidase K** in 10mM phosphate buffer, pH 7.4 at 30 °C). The dipeptide Ac-[2]Abz-Gly-OH was used as a reference signal. Measurements were performed in triplicate.

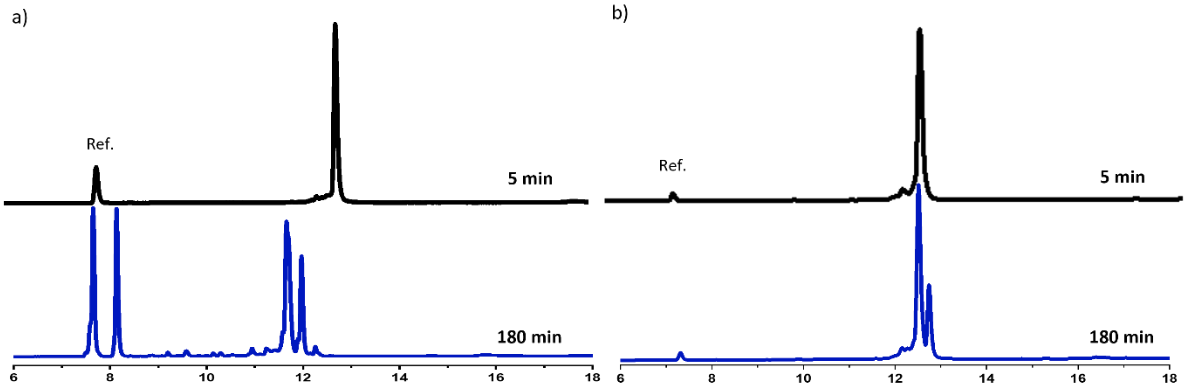

Figure S3: Real-time monitoring of proteolytic digestion of 5-180 min (HPLC, DAD-280 nm) of a) **SAJO-β** (100 µM peptide concentration) and b) **SAJO-2β** (100 µM peptide concentration) during incubation with **β-trypsin** (20 µM concentration) at 30 °C. The dipeptide Ac-[2]Abz-Gly-OH was used as a reference signal. Measurements were performed in triplicate.

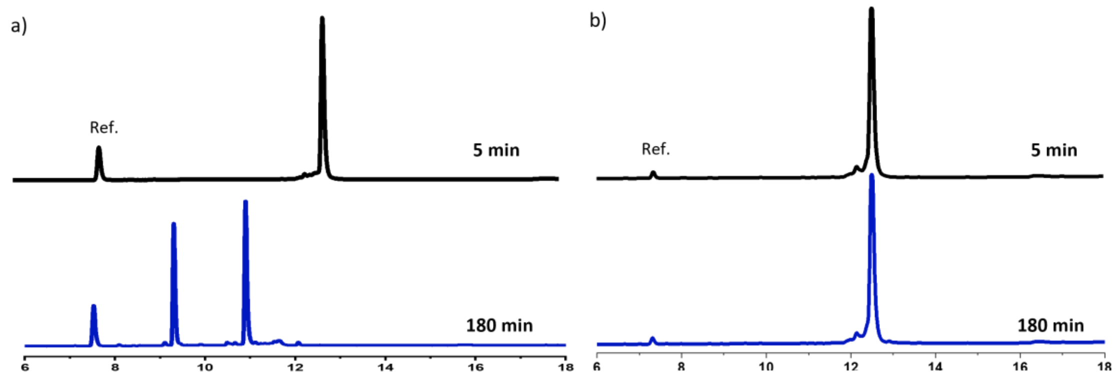

Figure S4: Real-time monitoring of proteolytic digestion of 5-180 min (HPLC, DAD-280 nm) of a) **SAJO-β** (100 µM peptide concentration) and b) **SAJO-2β** (100 µM peptide concentration) during incubation with **α-chymotrypsin** (20 µM concentration) at 30 °C. The dipeptide Ac-[2]Abz-Gly-OH was used as a reference signal. Measurements were performed in triplicate.

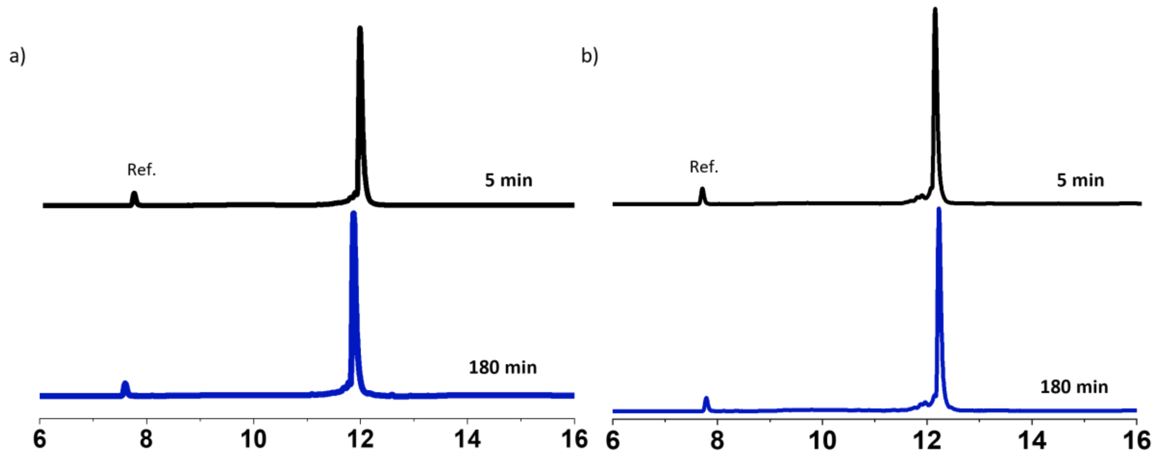

Figure S5: Real-time monitoring of proteolytic digestion of 5-180 min (HPLC, DAD-280 nm) of a) **SAJO-β** (100 µM peptide concentration) and b) **SAJO-2β** (100 µM peptide concentration) during incubation with **Proteinase K** (20 µM concentration) at 30 °C. The dipeptide Ac-[2]Abz-Gly-OH was used as a reference signal. Measurements were performed in triplicate.

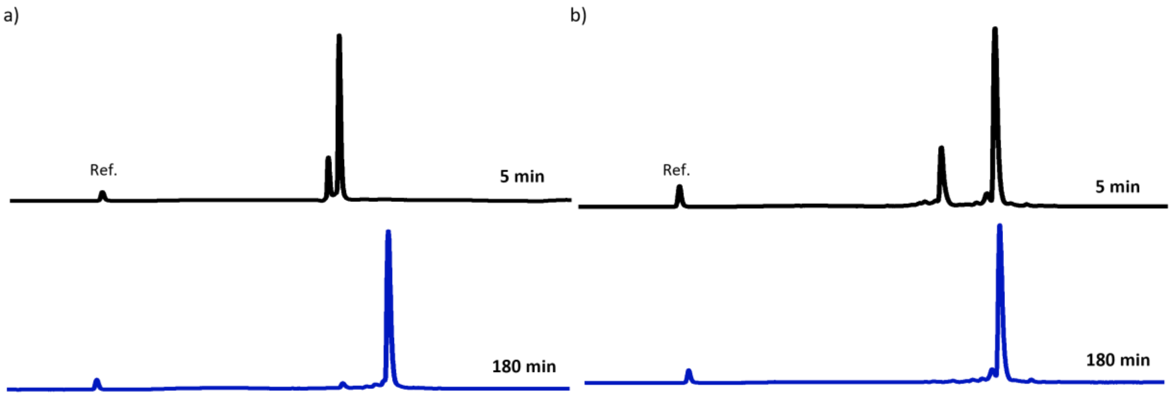

Figure S6: Real-time monitoring of proteolytic digestion of 5-180 min (HPLC, DAD-280 nm) of a) **SAJO-β** (100 µM peptide concentration) and b) **SAJO-2β** (100 µM peptide concentration) during incubation with **Carboxypeptidase B** (20 µM concentration) at 30 °C. The dipeptide Ac-[2]Abz-Gly-OH was used as a reference signal. Measurements were performed in triplicate.

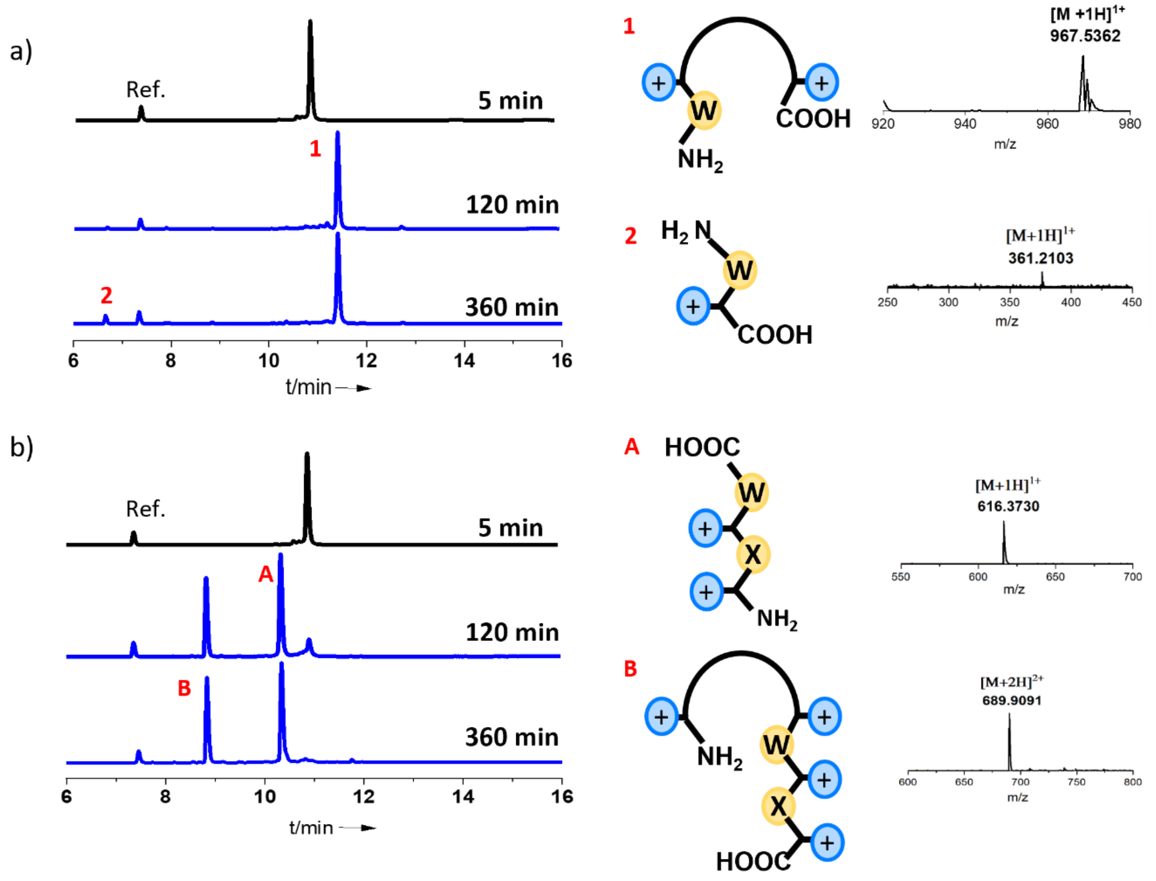

Figure S7: Real-time monitoring of proteolytic digestion of **SAJO-2** (100 µM peptide concentration)**,** 5-360 min (HPLC, DAD-280 nm) during incubation with a) **β-trypsin** (100 µM peptide concentration) and b) **α-chymotrypsin** (both 20 µM concentration) at 30 °C. The dipeptide Ac-[2]Abz-Gly-OH was used as a reference signal. Measurements were performed in triplicate according to a prior experimental procedure[1].

2. Lipid binding assay further data

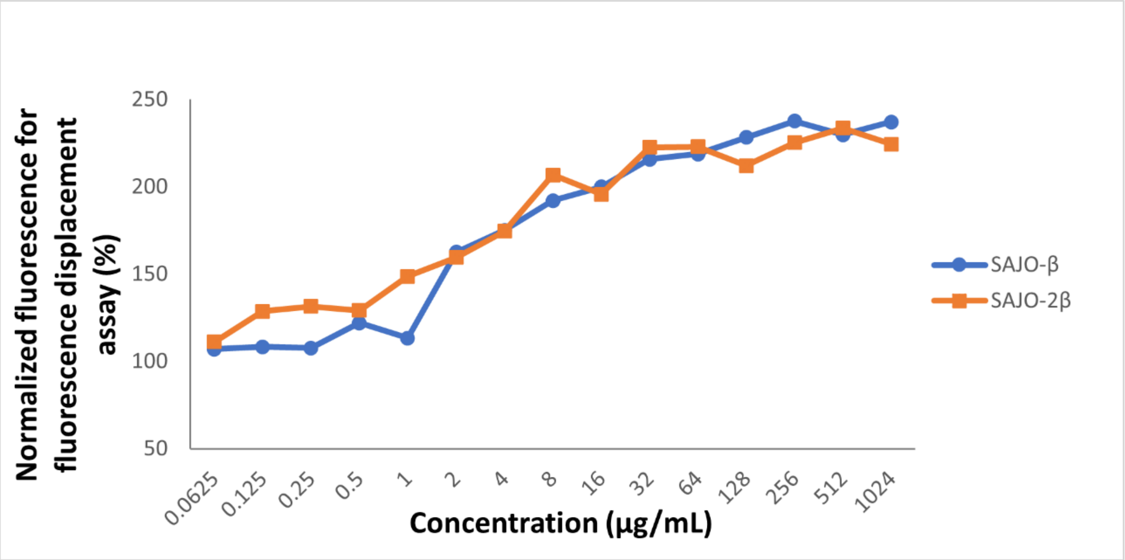

Figure S8: Lipid interaction of the **SAJO-β** and **SAJO-2β** detected *via* the release of Bodipy TR Cadaverine dye. Peptides in (two-fold serial dilutions, 0.0625 µg/mL to 1024 µg/mL) were incubated with lipopolysaccharides from Salmonella Typhimurium (7.5 µg/mL in Tris buffer) and Bodipy TR Cadaverine (2.1 µM in Tris buffer). All experiments were performed with three technical replicates with three independent measurements.

In **Figure S8**, a dose-dependent increase in fluorescence can be observed. As the concentration of peptides increases, the fluorescence displacement also increases. This suggests that both SAJO-β and SAJO-2β effectively displace the Bodipy TR cadaverine probe from LPS, implying binding to LPS. At higher concentrations, the fluorescence increase reached a plateau, suggesting the binding sites on LPS may be saturated.

3. Transmission electron microscopy images

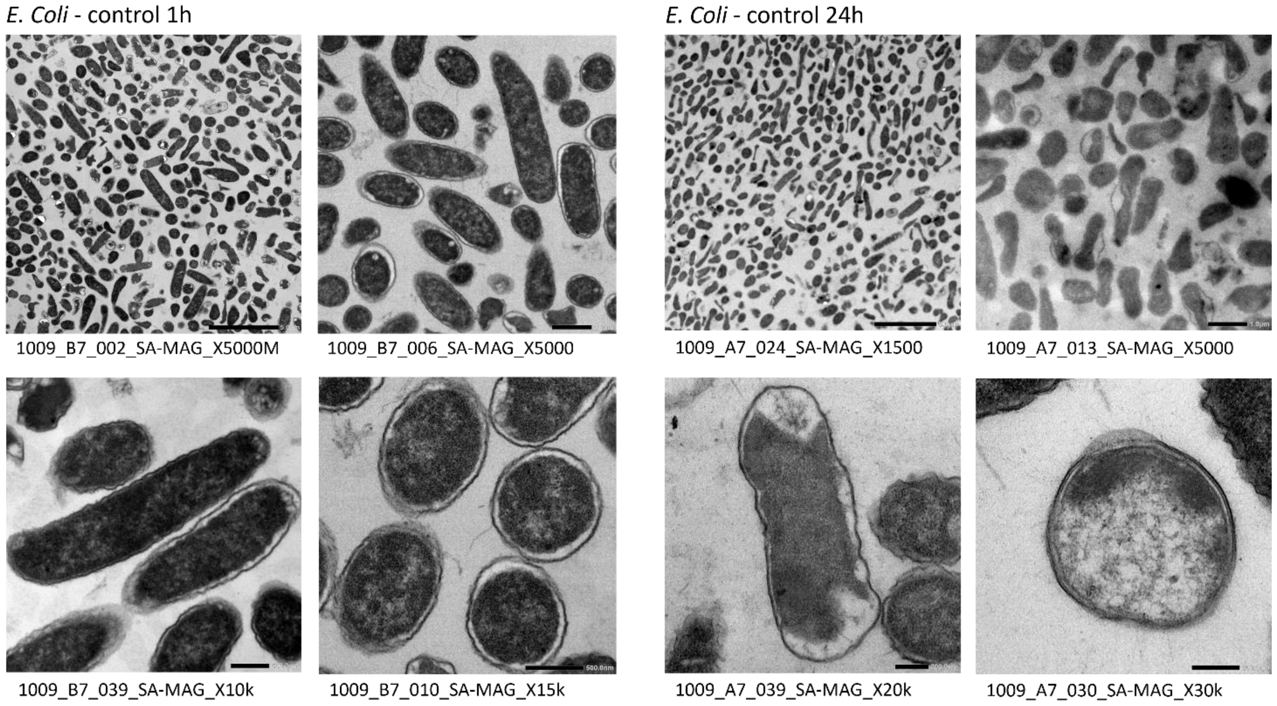

Figure S9: Negative control samples of untreated *E. coli* ATCC 25922

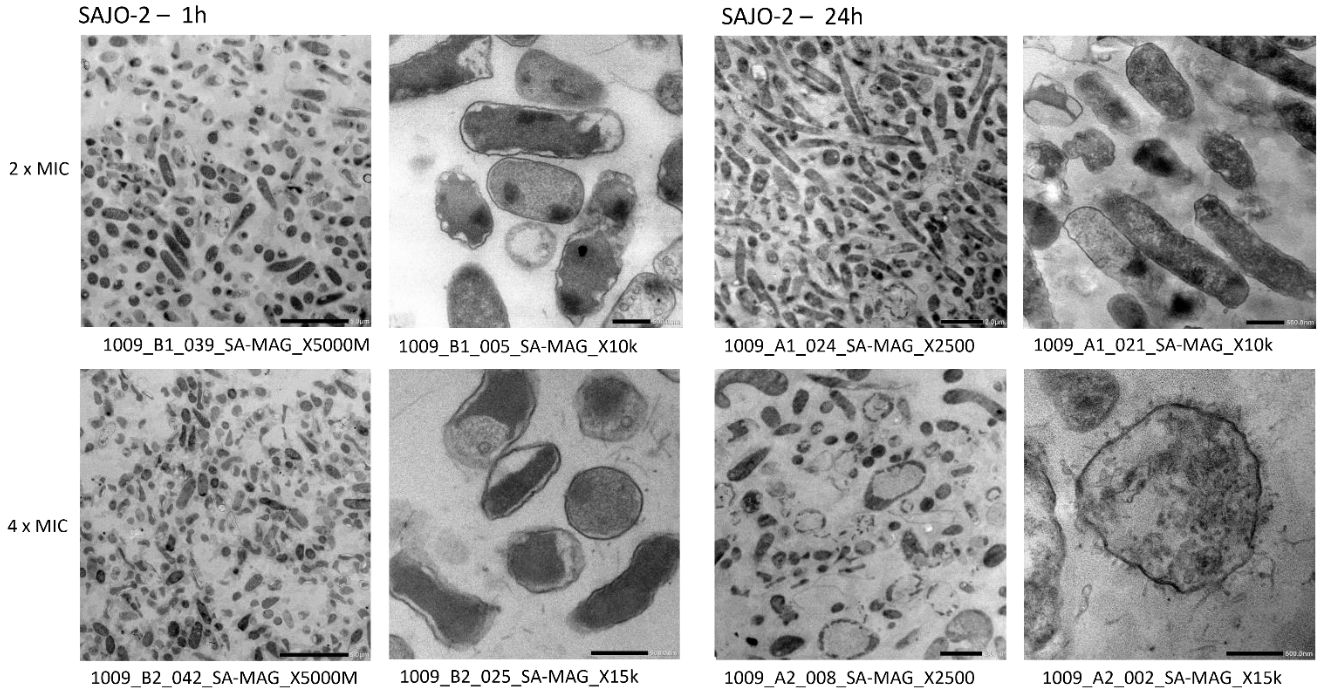

Figure S10: TEM images for SAJO-2 treated samples of *E. coli* ATCC 25922 after 1 h and 24 h of incubation at 37 °C. Top: 2 x MIC, bottom: 4 x MIC

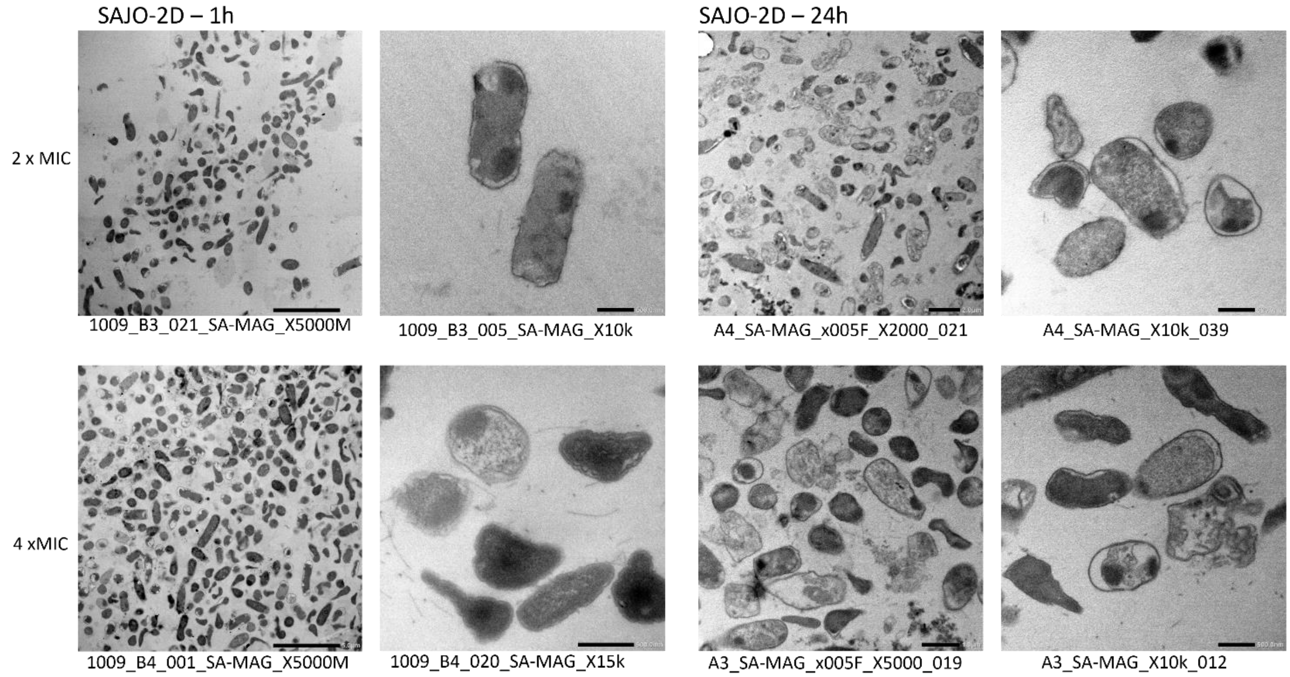

Figure S11: TEM images for SAJO-2D treated samples of *E. coli* ATCC 25922 after 1 h and 24 h of incubation at 37 °C. Top: 2 x MIC, bottom: 4 x MIC

4. Adaptive laboratory evolution further data

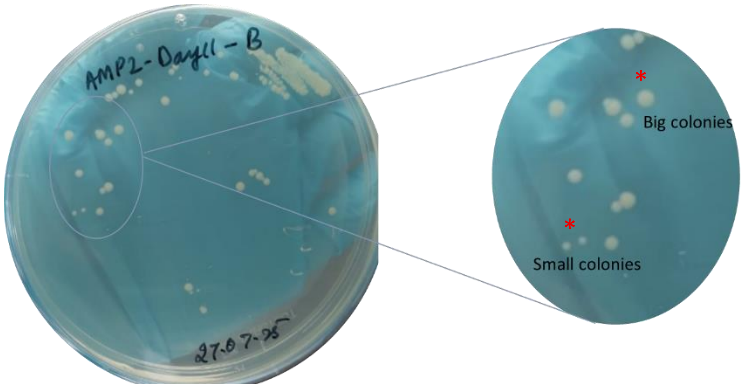

Figure S12: Representative image of al LB agar plate with a zoom-in view illustrating the colony morphology variations of the SAJO-2D evolved *E. coli* strain.

Table S2: Antimicrobial activities of SAJO-2 and SAJO-2D after six passages in peptide-free medium

	**1 * MIC (μg/mL)**			
Bacterial strains	SAJO-2		SAJO-2D	
	Before passaging	After passaging	Before passaging	After passaging
E. coli	16	16	16	16
SAJO-2_Day11_evolved strain H	256	32	-	-
SAJO-2D_Day11_evolved strain B (Small colonies)	-	-	1024	512
SAJO-2D_Day11_evolved strain B (Big colonies)	-	-	1024	32

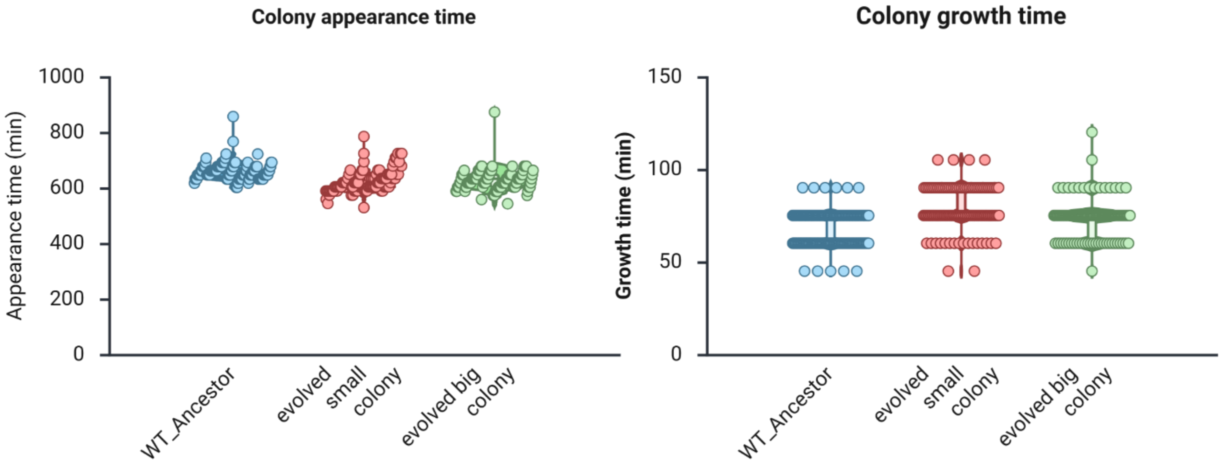

Figure S13: Scan-Lag data plotted for the small and big colonies of the SAJO-2D evolved E. coli strain and wild type plotted against appearance and growth time [min].

5. Molecular dynamic simulations further data

- Contact heat maps

a) **SAJO-2 – Membrane Heads Contact Hetamap (3 Å cutoff)**

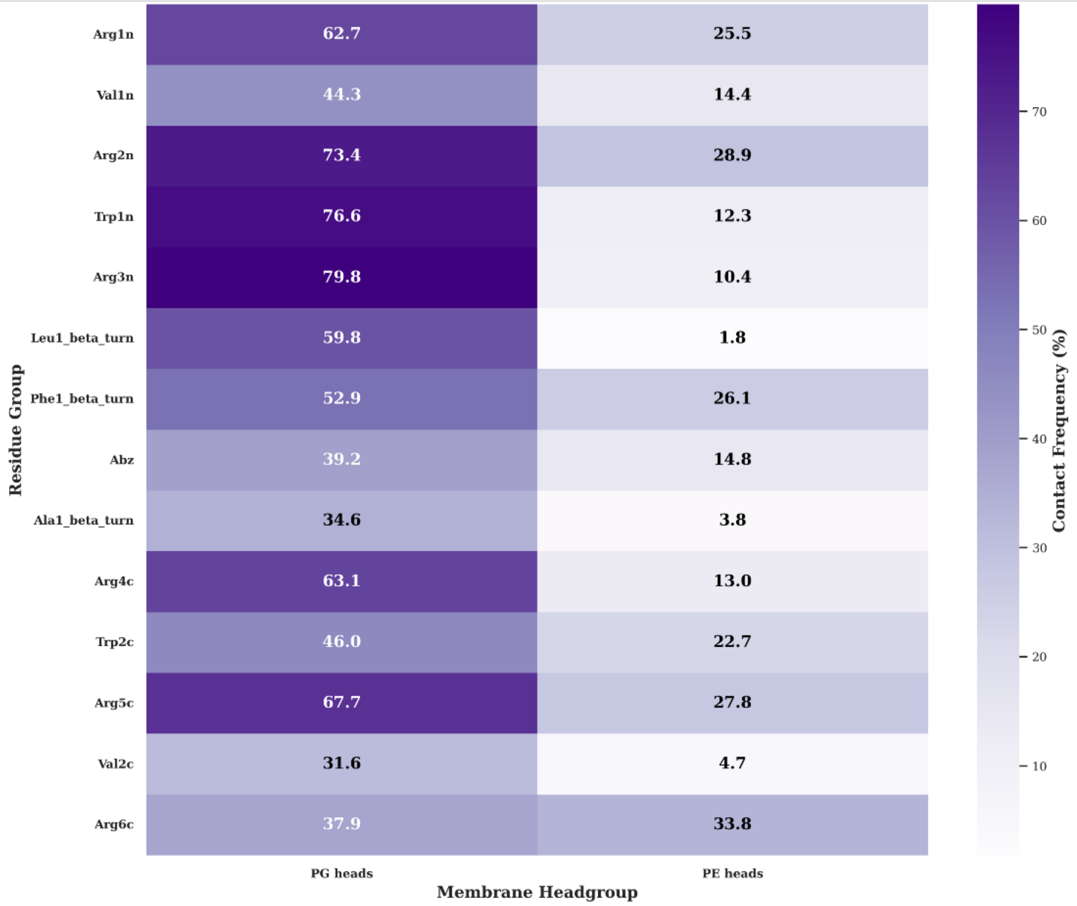

b)

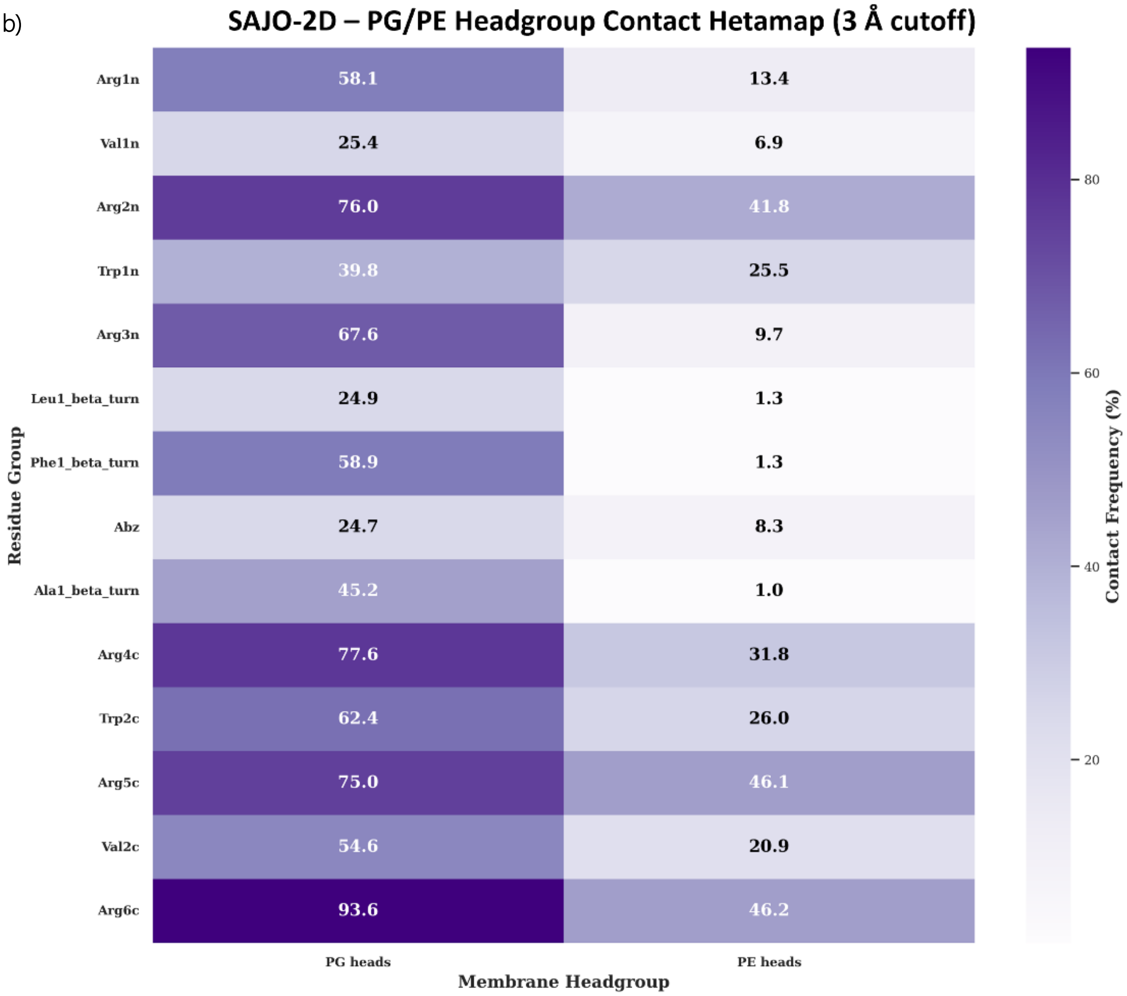
SAJO-2D – PG/PE Headgroup Contact Hetamap (3 Å cutoff)
Arg1n
Val1n
Arg2n
Trp1n
Arg3n
Leu1_beta_turn
Phe1_beta_turn
Abz
Ala1_beta_turn
Arg4c
Trp2c
Arg5c
Val2c
Arg6c
58.1
13.4
25.4
6.9
76.0
41.8
39.8
25.5
67.6
9.7
24.9
1.3
58.9
1.3
24.7
8.3
45.2
1.0
77.6
31.8
62.4
26.0
75.0
46.1
54.6
20.9
93.6
46.2
Residue Group
PG heads
PE heads
Membrane Headgroup
Contact Frequency (%)
80
60
40
20

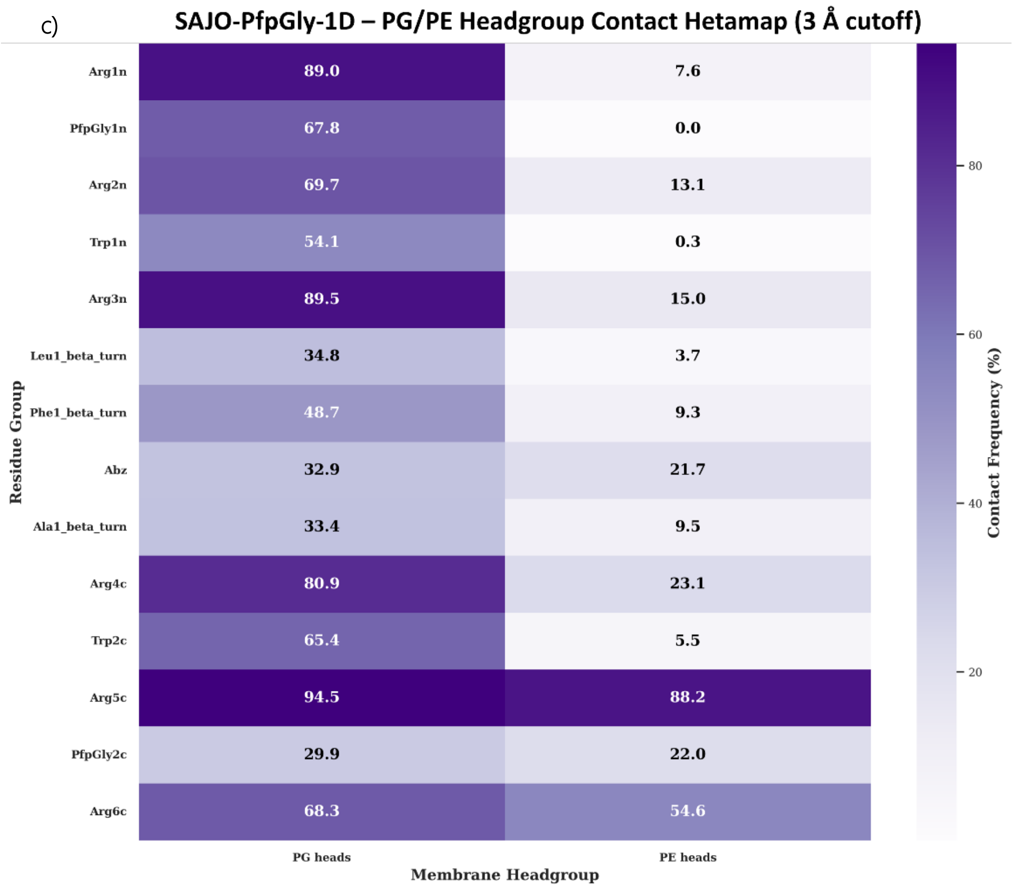

Figure S14: Contact heat maps showing residue-lipid headgroup interactions for (a) SAJO-2, (b) SAJO-2D, and (c) SAJO-PfpGly-1D. Colors represent the percentage of simulation frames in which each residue lies within 3 Å of the indicated lipid headgroup (white = low, dark violet = high). The maps illustrate residue-specific interaction strengths and selectivity toward POPE versus POPG.

- Insertion depth

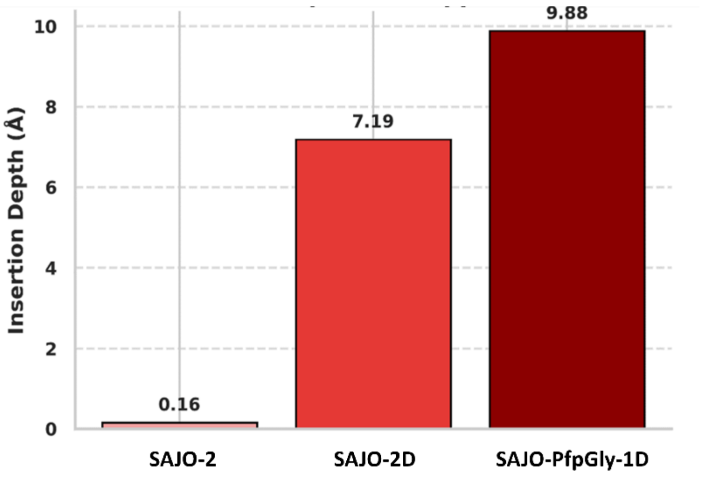

Figure S15: Insertion depth of SAJO-2, SAJO-2D and SAJO-PfpGly-1D

6. Synthesis and purification of peptides

Peptide synthesis was carried out utilizing a Liberty Blue automated microwave-assisted peptide synthesizer (CEM Corporation, Matthews, NC, USA) through an Fmoc-based solid-phase peptides synthesis (SPPS) approach. **SAJO-2, SAJO-D, SAJO-1D, SAJO-2D, SAJO-PfpGly-1D, SAJO-β** and **SAJO-2β** were synthesized as C-terminal acids on a Cl-MPA ProTide LL resin (0.17 mmol/g resin substitution) and Fmoc-Arg(Pbf) Wang resin (0.25-0.27 mmol/g resin substitution) at a 0.1 mmol scale. All amino acids were coupled using Oxyma/DIC as the activators. Fmoc removal was performed with 20 % piperidine in DMF. The non-standard amino acid PfpGly employed a unique coupling process that uses 1.5 equivalents of amino acid and extended microwave heating duration with extra washing stages. All peptides were released from the resin by exposure to a solution of TFA/TIPS/H2O (90/5/5) for 3 h at ambient temperature. The peptides were rinsed with TFA/DCM, the organic solvent was eliminated, and the peptides were precipitated using cold diethyl ether. HPLC purification resulted in the isolation of approximately 25-35 mg of the pure peptide. Table S3 presents the general HPLC conditions for purifying peptides.

Table S3: Gradient information for investigating the purity of synthesized, D/β amino acid peptide variants on an analytical HPLC. Eluents: Solvent A = 0.1% (v/v) TFA in water, Solvent B = 0.1% (v/v) TFA in ACN

Time [min]	Solvent A [%]	Solvent B [%]	Flow rate [mL/min]
0.0	90.0	10.0	1.00
18.0	30.0	70.0	1.00
19.0	0.0	100.0	1.00
21.0	0.0	100.0	1.00
22.0	90.0	10.0	1.00
25.0	90.0	10.0	1.00

Table S4: Library of peptides synthesized with the incorporation of non-canonical amino acids

Peptide	Sequence
SAJO-2	Arg-Val-Arg-Trp-Arg-D-Leu-[D-Phe-2-Abz]-D-Ala-Arg-Trp-Arg-Val-Arg
SAJO-2LD	D-Arg-D-Val-D-Arg-D-Trp-D-Arg-D-Leu-[D-Phe-2-Abz]-D-Ala-D-Arg-D-Trp-D-Arg-D-Val-D-Arg
SAJO-D	D-Arg-Val-D-Arg-Trp-D-Arg-D-Leu-[D-Phe-2-Abz]-D-Ala-D-Arg-Trp-D-Arg-Val-D-Arg
SAJO-1D	Arg-Val-D-Arg-Trp-Arg-D-Leu-[D-Phe-2-Abz]-D-Ala-Arg-Trp-D-Arg-Val-Arg
SAJO-2D	Arg-Val-D-Arg-Trp-D-Arg-D-Leu-[D-Phe-2-Abz]-D-Ala-D-Arg-Trp-D-Arg-Val-Arg
SAJO-PfpGly-1D	Arg-PfpGly-D-Arg-Trp-Arg-D-Leu-[D-Phe-2-Abz]-D-Ala-Arg-Trp-D-Arg-PfpGly-Arg
SAJO-β	Arg-Val-βhomoArg-Trp-Arg-D-Leu-[D-Phe-2-Abz]-D-Ala-Arg-Trp-βhomoArg-Val-Arg
SAJO-2β	Arg-Val-βhomoArg-Trp-βhomoArg-D-Leu-[D-Phe-2-Abz]-D-Ala-βhomoArg-Trp-βhomoArg-Val-Arg

6.1 SAJO-2

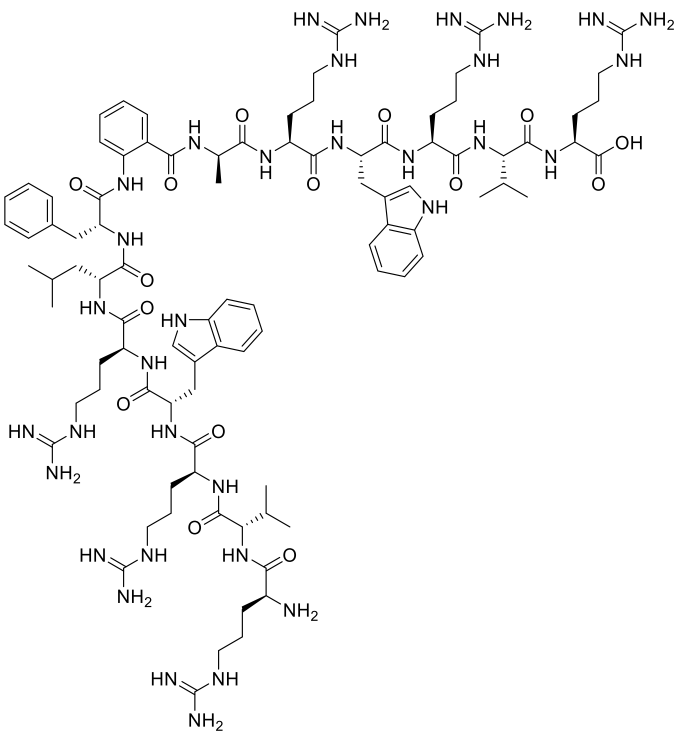

Figure S16: Chemical structure of **SAJO-2**

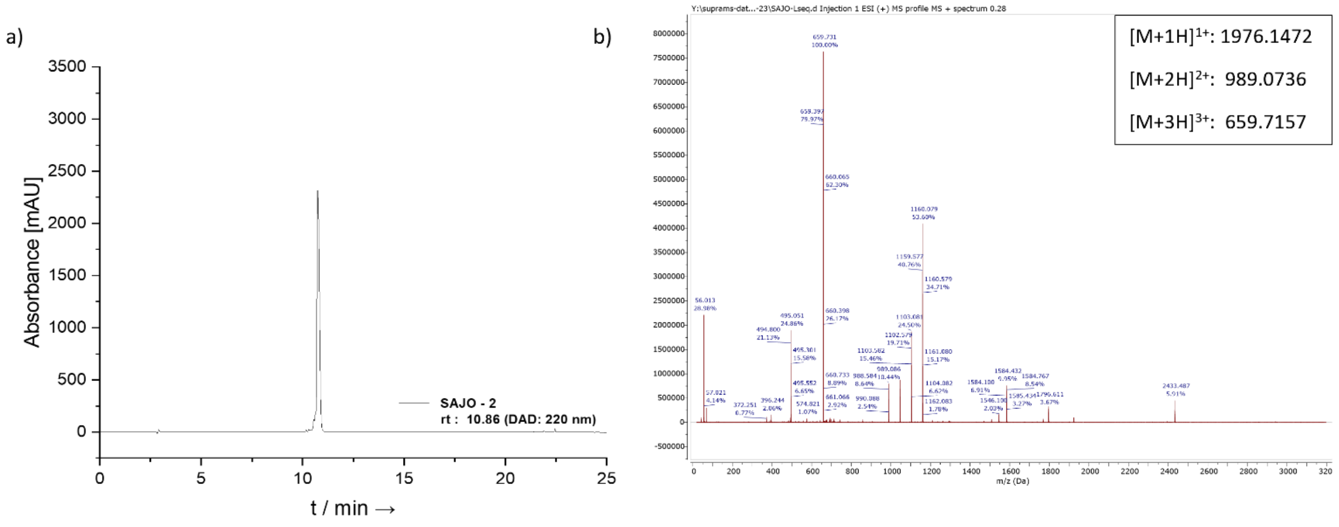

Figure S17: **a)** HPLC chromatogram of the purified peptide **SAJO-2** with DAD detection at 220 nm, solvent: **(A)** H_2O + 0.1% TFA / **(B)** ACN + 0.1% TFA / gradient: 10% (B) → 70% (B) over 18 min. **b)** High resolution mass spectrometry (HRMS) spectrum of **SAJO-2** in positive ionization mode. Calculated ion species $[M + H]^+$ for this peptide are listed in the spectral data.

6.2 SAJO-D

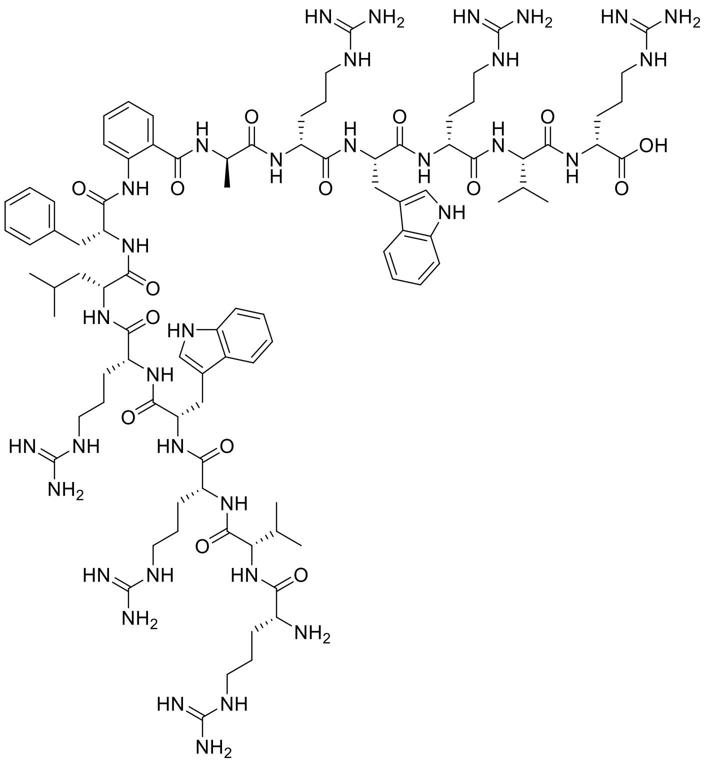

Figure S18: Chemical structure of **SAJO-D**

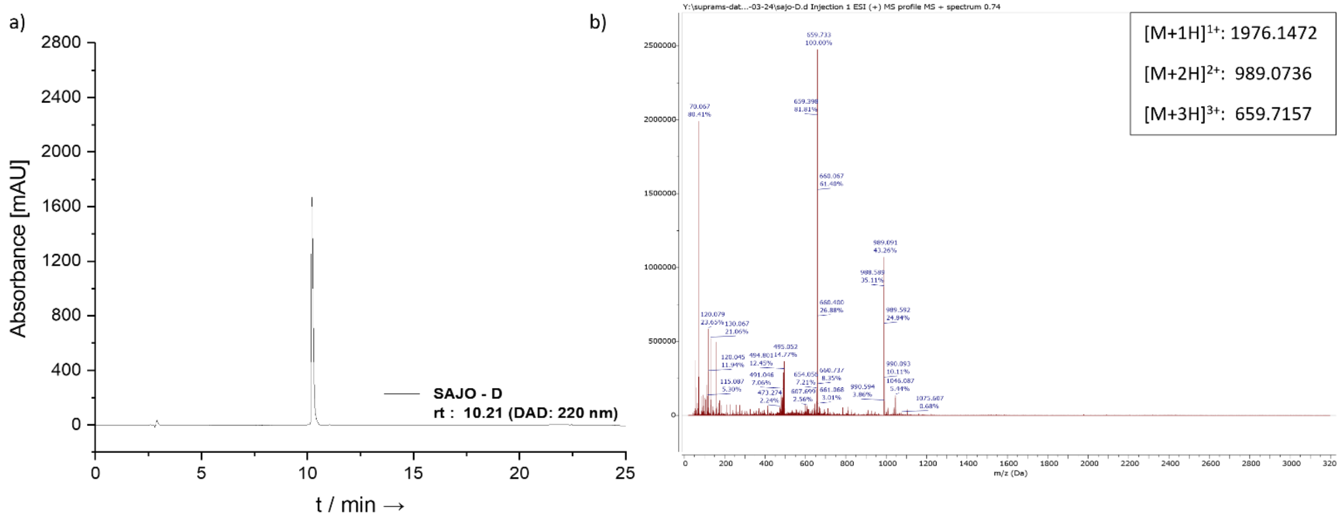

Figure S19: a) HPLC chromatogram of the purified peptide **SAJO-D** with DAD detection at 220 nm, solvent: **(A)** H_2O + 0.1% TFA / **(B)** ACN + 0.1% TFA / gradient: 10% (B) → 70% (B) over 18 min. **b)** High resolution mass spectrometry (HRMS) spectrum of **SAJO-D** in positive ionization mode. Calculated ion species $[M + H]^+$ for this peptide are listed in the spectral data.

6.3 SAJO-1D

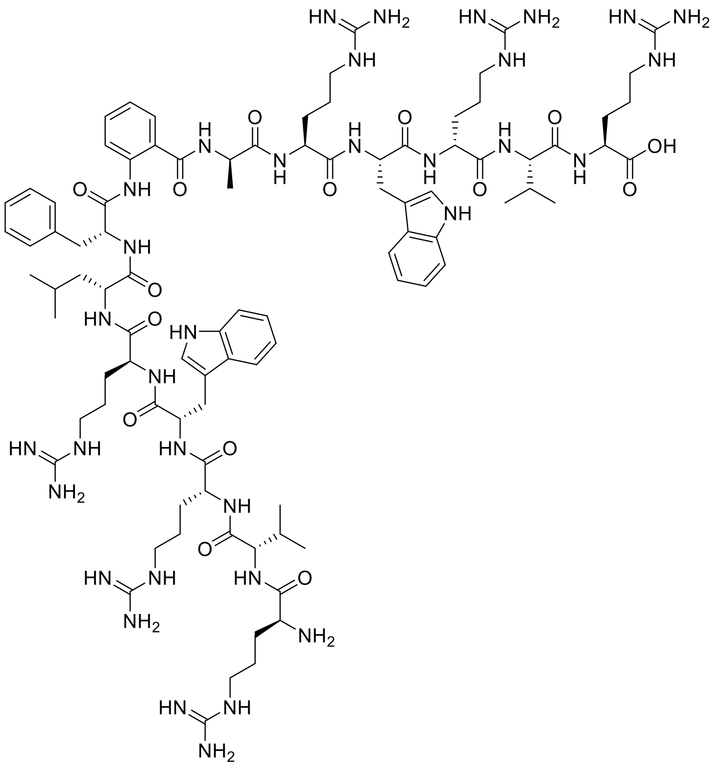

Figure S20: Chemical structure of **SAJO-1D**

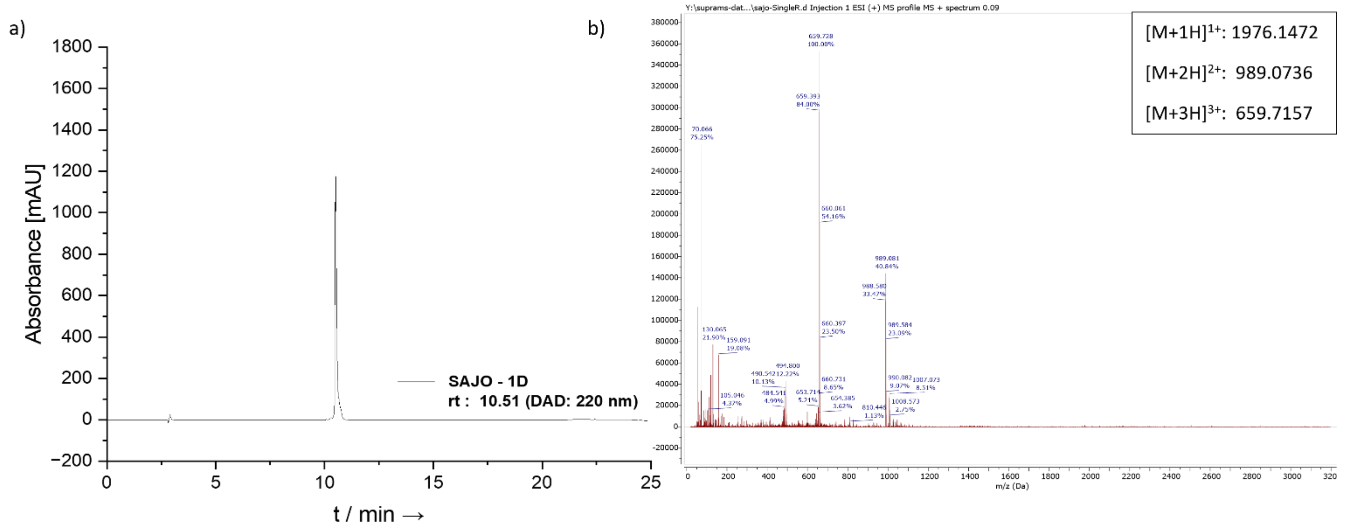

Figure S21: a) HPLC chromatogram of the purified peptide **SAJO-1D** with DAD detection at 220 nm, solvent: **(A)** H_2O + 0.1% TFA / **(B)** ACN + 0.1% TFA / gradient: 10% (B) → 70% (B) over 18 min. **b)** High resolution mass spectrometry (HRMS) spectrum of **SAJO-1D** in positive ionization mode. Calculated ion species $[M + H]^+$ for this peptide are listed in the spectral data.

6.4 SAJO-2D

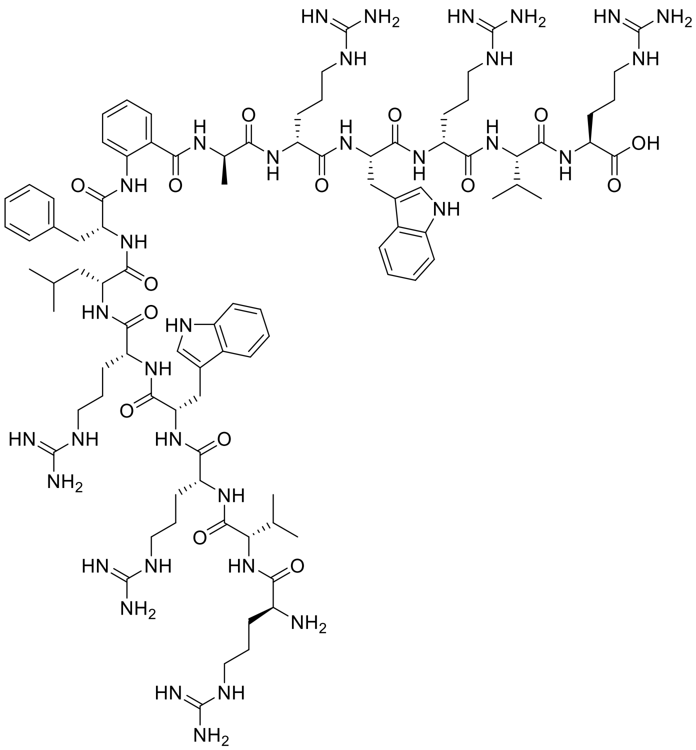

Figure S22: Chemical structure of **SAJO-2D**

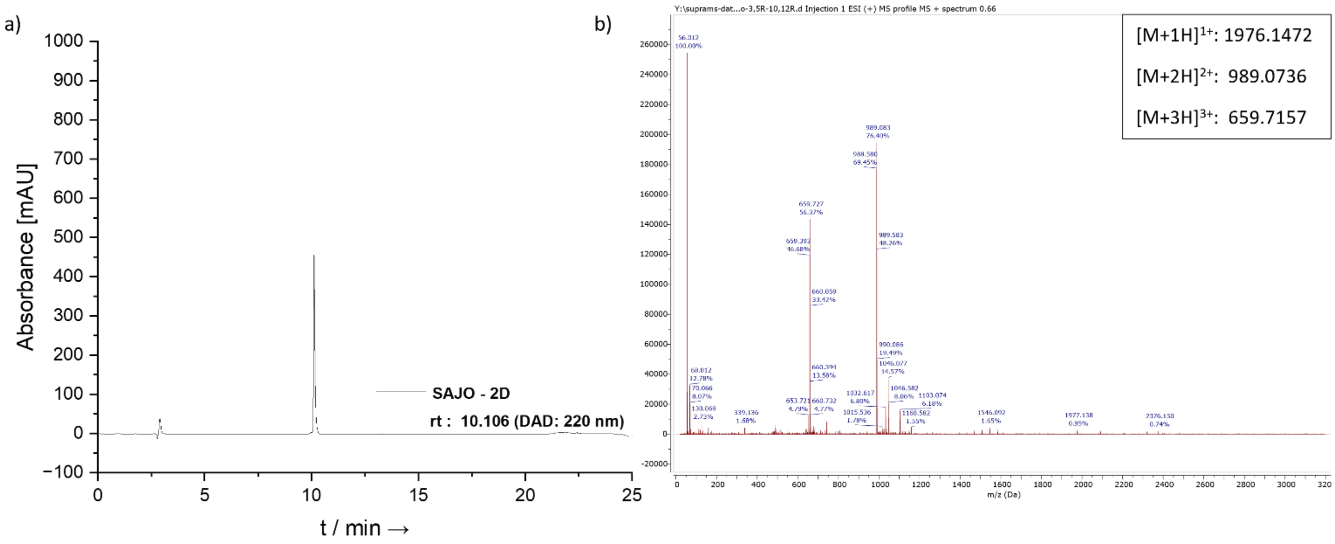

Figure S23: a) HPLC chromatogram of the purified peptide **SAJO-2D** with DAD detection at 220 nm, solvent: **(A)** H_2O + 0.1% TFA / **(B)** ACN + 0.1% TFA / gradient: 10% (B) → 70% (B) over 18 min. **b)** High resolution mass spectrometry (HRMS) spectrum of **SAJO-2D** in positive ionization mode. Calculated ion species $[M + H]^+$ for this peptide are listed in the spectral data.

6.5 SAJO-PfpGly-1D

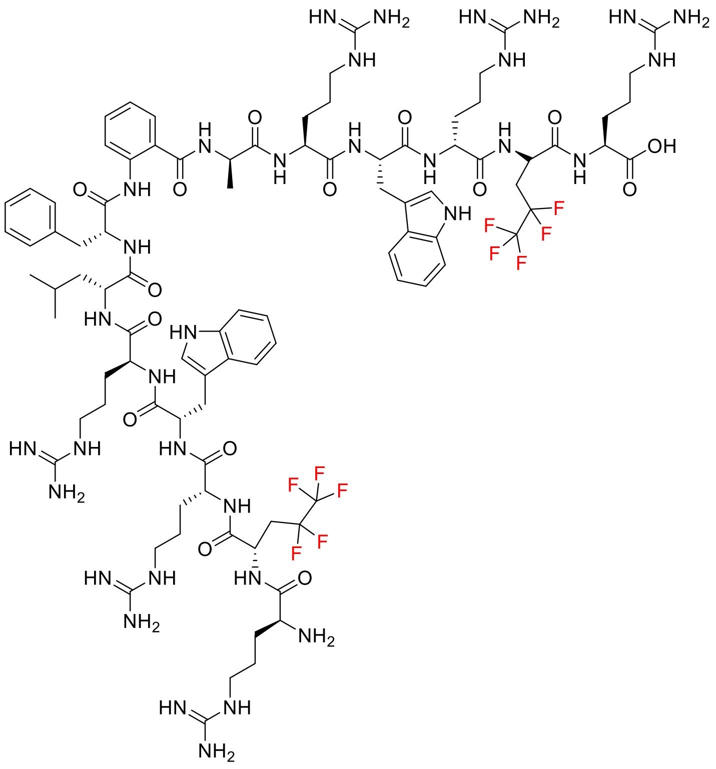

Figure S24: Chemical structure of SAJO-PfpGly-1D

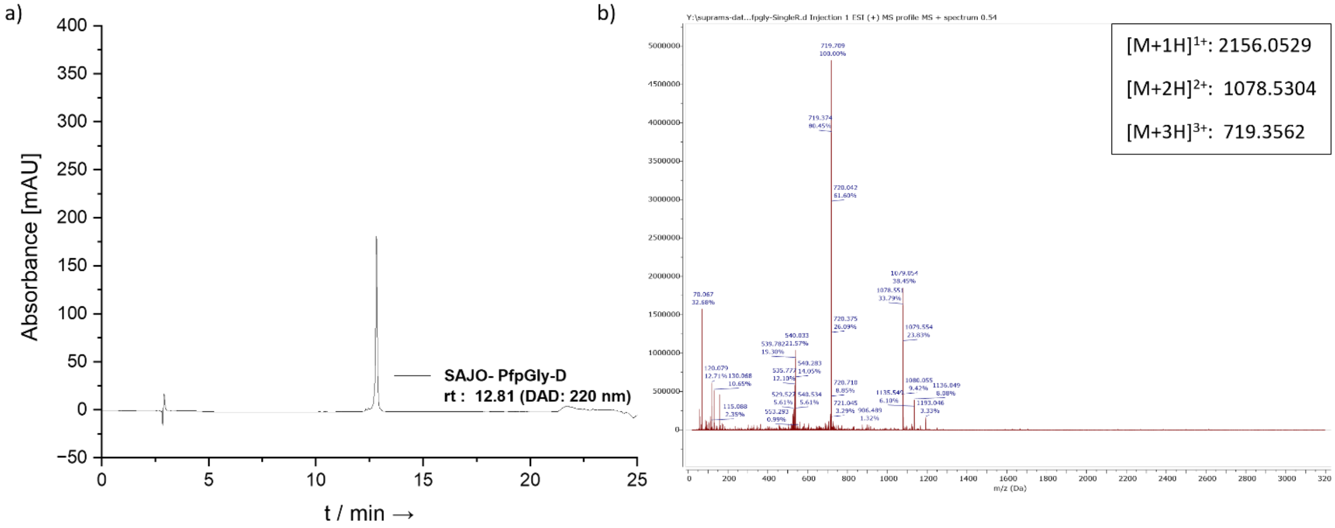

Figure S25: a) HPLC chromatogram of the purified peptide **SAJO-PfpGly-1D** with DAD detection at 220 nm, solvent: **(A)** H_2O + 0.1% TFA / **(B)** ACN + 0.1% TFA / gradient: 10% (B) → 60% (B) over 18 min. **b)** High resolution mass spectrometry (HRMS) spectrum of **SAJO-PfpGly-1D** in positive ionization mode. Calculated ion species $[M + H]^+$ for this peptide are listed in the spectral data.

6.6 SAJO-β

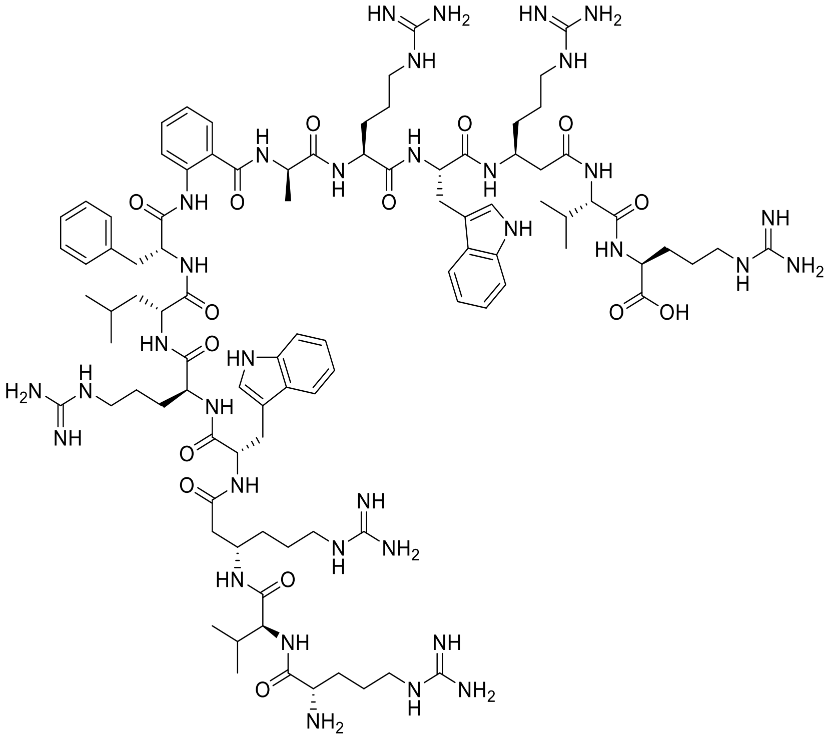

Figure S26: Chemical structure of **SAJO-β**

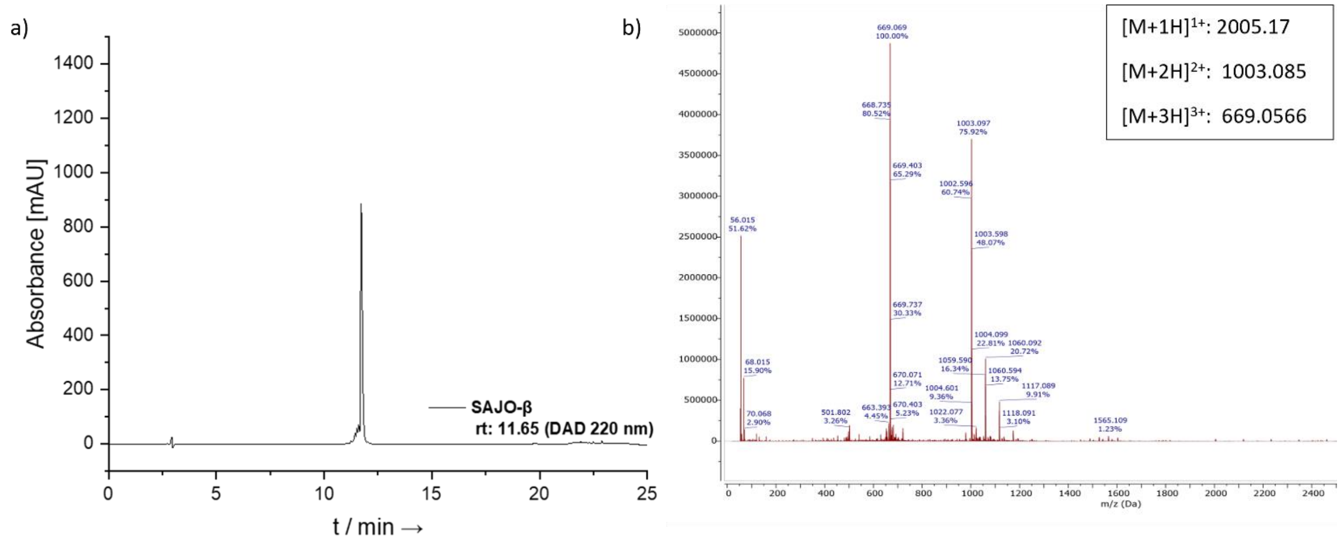

Figure S27: a) HPLC chromatogram of the purified peptide **SAJO-β** with DAD detection at 220 nm, solvent: **(A)** H_2O + 0.1% TFA / **(B)** ACN + 0.1% TFA / gradient: 10% (B) → 60% (B) over 18 min. **b)** High resolution mass spectrometry (HRMS) spectrum of **SAJO-β** in positive ionization mode. Calculated ion species $[M + H]^+$ for this peptide are listed in the spectral data.

6.7 SAJO-2β

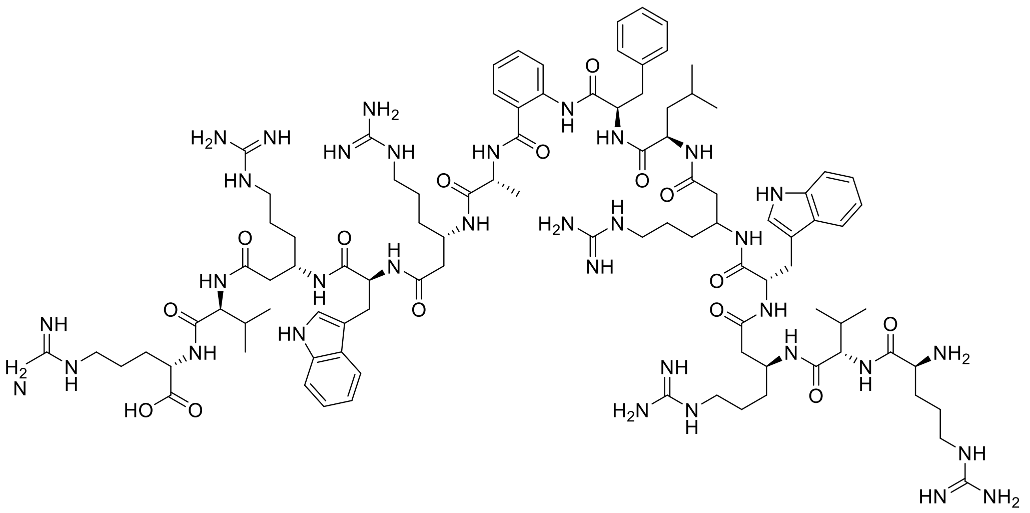

Figure S28: Chemical structure of **SAJO-2β**

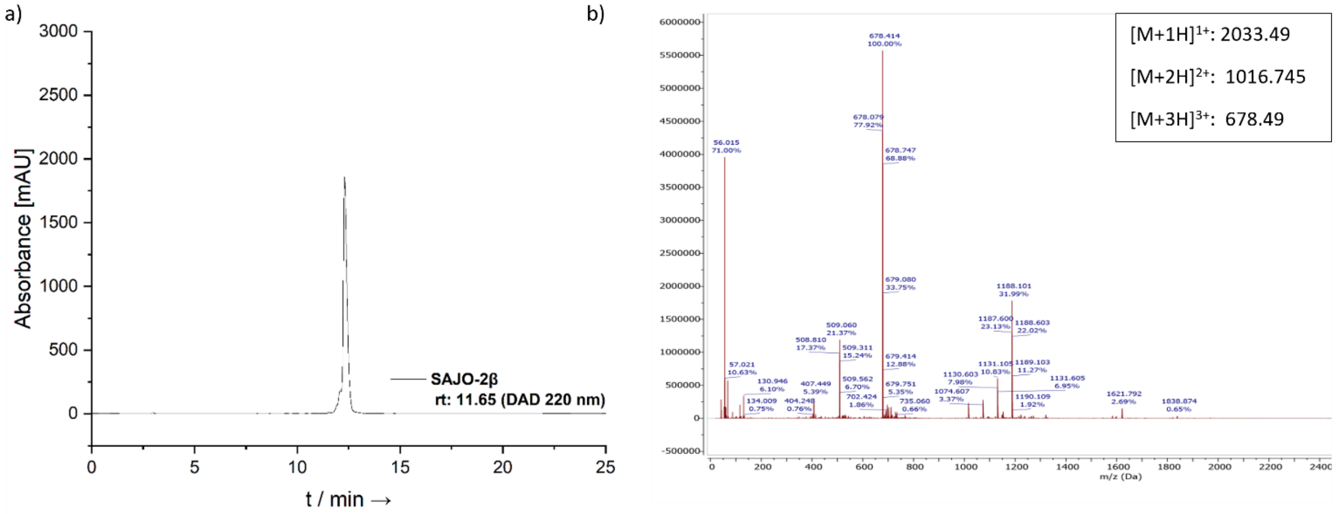

Figure S29: a) HPLC chromatogram of the purified peptide **SAJO-2β** with DAD detection at 220 nm, solvent: **(A)** H_2O + 0.1% TFA / **(B)** ACN + 0.1% TFA / gradient: 10% (B) → 60% (B) over 18 min. **b)** High resolution mass spectrometry (HRMS) spectrum of **SAJO-2β** in positive ionization mode. Calculated ion species $[M + H]^+$ for this peptide are listed in the spectral data.

7. References

(1) Chowdhary, S.; Pelzer, T.; Saathoff, M.; Quaas, E.; Pendl, J.; Fulde, M.; Koksch, B. Fine-tuning the Antimicrobial Activity of Β-hairpin Peptides with Fluorinated Amino Acids. *Pept. Sci.* **2023**, *115* (3), e24306. https://doi.org/10.1002/pep2.24306.
